# Recent Progress of Nanogenerators for Green Energy Harvesting: Performance, Applications, and Challenges

**DOI:** 10.3390/nano12152549

**Published:** 2022-07-25

**Authors:** Enrique Delgado-Alvarado, Ernesto A. Elvira-Hernández, José Hernández-Hernández, Jesús Huerta-Chua, Héctor Vázquez-Leal, Jaime Martínez-Castillo, Pedro J. García-Ramírez, Agustín L. Herrera-May

**Affiliations:** 1 Micro and Nanotechnology Research Center, Universidad Veracruzana, Boca del Río 94294, VER, Mexico; endelgado@uv.mx (E.D.-A.); aelvirah@hotmail.com (E.A.E.-H.); jaimartinez@uv.mx (J.M.-C.); 2 Departamento de Investigación, Instituto Tecnológico Superior de Poza Rica, Tecnológico Nacional de México, Poza Riza 93230, VER, Mexico; chua@itspozarica.edu.mx; 3 Facultad de Ingeniería Mecánica y Ciencias Navales, Universidad Veracruzana, Boca del Río 94294, VER, Mexico; 4 Maestría en Ingeniería Aplicada, Facultad de Ingeniería de la Construcción y el Hábitat, Universidad Veracruzana, Boca del Río 94294, VER, Mexico; 5 Facultad de Instrumentación Electrónica, Universidad Veracruzana, Boca del Río 94294, VER, Mexico; hvazquez@uv.mx; 6 Instituto de Ingeniería, Universidad Veracruzana, Boca del Río 94294, VER, Mexico; jagarcia@uv.mx

**Keywords:** energy harvesting, green energy, hybrid nanogenerators, piezoelectric nanogenerator, thermoelectric nanogenerators, triboelectric nanogenerator

## Abstract

Natural sources of green energy include sunshine, water, biomass, geothermal heat, and wind. These energies are alternate forms of electrical energy that do not rely on fossil fuels. Green energy is environmentally benign, as it avoids the generation of greenhouse gases and pollutants. Various systems and equipment have been utilized to gather natural energy. However, most technologies need a huge amount of infrastructure and expensive equipment in order to power electronic gadgets, smart sensors, and wearable devices. Nanogenerators have recently emerged as an alternative technique for collecting energy from both natural and artificial sources, with significant benefits such as light weight, low-cost production, simple operation, easy signal processing, and low-cost materials. These nanogenerators might power electronic components and wearable devices used in a variety of applications such as telecommunications, the medical sector, the military and automotive industries, and internet of things (IoT) devices. We describe new research on the performance of nanogenerators employing several green energy acquisition processes such as piezoelectric, electromagnetic, thermoelectric, and triboelectric. Furthermore, the materials, applications, challenges, and future prospects of several nanogenerators are discussed.

## 1. Introduction

The internet of things (IoT) gadgets, smart sensors, internet of medical things (IoMT) for healthcare systems, and consumer electronics devices have seen significant expansion in recent years. These devices often employ traditional batteries, which have drawbacks owing to their huge size, finite lifetime, and harmful components that contaminate the environment [1,2,3]. This issue with traditional batteries may restrict the efficiency of future IoT gadgets, smart sensors, and wearable devices. Thus, new eco-friendly alternative technologies to power these gadgets are current and future research challenges. Recent studies [4,5,6,7,8] have described nanogenerators capable of harvesting green energy by several transduction methods such as the piezoelectric, triboelectric, electromagnetic, and thermoelectric effects. The nanogenerators can harvest green energy from natural and artificial sources from wind, water, thermal, solar, mechanical vibrations, and motions of the human body [9,10,11,12,13]. These nanogenerators have unique features such as light weight, low-cost fabrication, tiny size, simple performance and signal processing, high power density, and a longer lifetime when compared to conventional batteries. Thus, nanogenerators provide a cost-effective alternative for powering future IoT devices, smart sensors, and consumer electronics products based on green energy harvesting from the environment. Furthermore, nanogenerators may be used to drive self-powered sensors for applications ranging from telecommunications to health monitoring, the automotive and military industries, agriculture, aerospace, and smart cities [14,15,16,17,18,19].

Most commercial low-power electronic devices require rectifier circuits to convert the variable output current of nanogenerators into direct current (DC). In addition, several researchers have used rectifiers coupled with antennas to design rectennas, which can harvest radio frequency (RF) energy and convert it to direct current [20,21,22,23,24,25]. Supercapacitors can also be integrated into nanogenerators to store their output power [26,27]. Thus, rectifier circuits and supercapacitors can enable the nanogenerators to have a consistent output power. In addition, hybrid nanogenerators may gather multiple green energy sources using two or more acquisition processes [28,29,30,31]. Due to this performance characteristic, hybrid nanogenerators can increase their output power densities in comparison to a single nanogenerator. The hybrid nanogenerators can power electronic devices for longer periods of time by utilizing various green energy sources (e.g., wind, heat, rain, solar radiation, and mechanical vibrations). These hybrid nanogenerators may be capable of harvesting a mix of green energies to continuously power electronics and sensors. This might enable the conversion of accessible green energy sources into electricity both during the day and at night, as well as in both indoor and outdoor environments.

More research is needed to increase the performance, stability, and reliability of nanogenerators. For instance, optimization methods may be utilized in the design of nano-generators for each individual application to forecast the best electrical and structural configurations and material selection. This optimized nanogenerator design can increase output power density and service time. Another idea is to employ wearable and flexible materials to create nanogenerators that are adaptive to the human body and gather biomechanical energy [32,33]. Additionally, effective packaging solutions for nanogenerators are necessary to improve their wear resistance and resistance to high temperature and humidity fluctuations. Better packing materials and the usage of long-lasting materials for nanogenerators can improve their reliability. The sensitivity of rectification circuits used in nanogenerators can be improved in the electronic section to produce a higher output DC power. Furthermore, these circuits may be manufactured utilizing microelectronic technology to reduce their size [34,35].

We present new research on nanogenerators that transform various green energy sources into electricity. This review looks at the principles of operation, materials, performance, and applications of several nanogenerators, including multiple green energy acquisition processes. The performance advantages of hybrid nanogenerators are also explored. We also consider the problems and views of nanogenerators, including their design phase, materials, energy storage, fabrication method, and dependability. Nanogenerators technology is an alternative solution for replacing traditional batteries and powering future electronic devices and sensors in the IoT and military industries, IoMT for healthcare systems, consumer electronics, telecommunications, automotive sector, robotics, wearable optoelectronics, and other fields.

## 2. Operation Principle

### 2.1. Vibration Energy

The vibration energy from the environment can be harvested using nanogenerators with transduction mechanisms such as piezoelectric, electromagnetic, triboelectric, and piezotronic effects. For instance, these nanogenerator types can convert mechanical vibrations caused by the wind effect, sound, water waves, human body motion, machines, and vehicles into electrical energy.

#### 2.1.1. Piezoelectric Nanogenerators

The piezoelectric nanogenerators (PENGs) use the piezoelectric effect to capture green energy from ocean water waves, wind, biomechanical movements, and environmental mechanical vibrations. The output voltage of this type of nanogenerator is affected by mechanical deformations and the parameters of its piezoelectric layer. Mechanical vibrations in the environment can induce varied deformations in the piezoelectric nano-generators that generate the AC output voltage. A piezoelectric layer, a substrate, and two electrodes make up these nanogenerators. PENGs feature a basic structural design, easy performance, a simple construction method, high stability, and a low cost [36,37,38,39,40,41,42,43,44].

#### 2.1.2. Electromagnetic Nanogenerators

Electromagnetic generators (EMGs) employ magnetic materials and coils to function according to the Faraday law. These generators may convert the kinetic energy of flowing water into electricity [45]. This wave flow is utilized to vary the location of the magnet material relative to the coil, resulting in a changing magnetic field that induces a voltage in the coil. However, as compared to triboelectric nanogenerators, these generators can have a larger volume and weight. Furthermore, EMGs require support structures that let them float on the water’s surface [46]. The performance of electromagnetic nanogenerators is determined by the rate of change of the magnetic flux. EMGs can be made to function at frequencies comparable to those of ocean waves to scavenge energy from them. Ocean waves move randomly at low frequencies of roughly 1 Hz [47]. The EMGs’ performance is limited by their low frequency. Due to wind sources and environmental mechanical vibrations, which may function at higher frequencies, EMGs are ideal for scavenging green energy.

#### 2.1.3. Triboelectricity Nanogenerators

Triboelectric nanogenerators (TENGs) may gather green energy from irregular surroundings at low frequencies by connecting contact electrification with electrostatic induction. Blue energy, for example, may be extracted from ocean wave motion, which is fundamentally random and travels at low frequencies (near to 1 Hz) [48,49,50,51,52,53,54,55,56,57,58,59,60]. The benefits of triboelectric nanogenerators are their small weight, low cost, simple operation principle, and lack of sophisticated production [61,62,63,64]. To attain the highest performance, the triboelectric materials and electro-mechanical designs of the nanogenerators must be optimized [65,66,67,68,69,70,71]. As a result, optimizing the design of triboelectric nanogenerators is critical for improving the conversion of green energy into electric energy.

**Figure 1:**
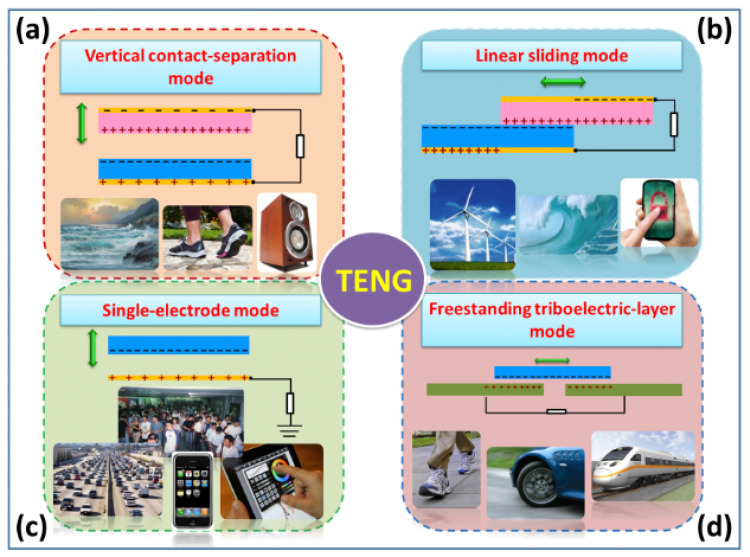
Several potential applications of TENGs using different operational modes: (**a**) vertical contact-separation, (**b**) linear sliding, (**c**) single-electrode, and (**d**) freestanding triboelectric-layer. Reprinted with permission from [72]. Copyright ©2014, Royal Society of Chemistry.

TENGs may be configured to function in four basic modes (Figure 1): vertical contact-separation (CS), lateral sliding (LS), single-electrode (SE), and freestanding triboelectric-layer (FSTL). TENGs usually require two triboelectric surfaces and two electrodes. Electron attraction between two triboelectric surfaces creates an electrostatic charge transfer from one surface to another in these operational modes. The displacement of the triboelectric layers changes their initial electrostatic state, resulting in an electric potential difference between the layers. The potential difference drives the current through the external load to balance the electrostatic state. The movement of the triboelectric layer in the opposite direction will generate a difference in the current flow. TENGs can therefore have alternating current (AC) voltages between their two output electrodes, depending on the triboelectric material type, operating mechanism, and green energy source.

#### 2.1.4. Piezotronic Nanogenerators

The piezotronic nanogenerators harvest low-frequency vibration/friction energy into electricity by using the linked piezoelectric and semiconducting capabilities of nanowires/nanobelts, as well as the influence of a Schottky barrier at the metal-semiconductor [73,74]. These nanogenerators might be incorporated into textile strands to recycle energy generated by human movement. Thus, the piezoelectronic nanogenerator is a potentially useful technology for harvesting/recycling energy from the environment to power self-powered nanodevices that may be operated wirelessly and remotely. This technique will enable self-powered wireless nanosystems and nanodevices to have a sustained energy supply [75].

### 2.2. Thermal Energy

Thermoelectric and pyroelectric nanogenerators can transform thermal energy from the environment into electrical energy to power electronic devices.

#### Thermoelectric and Pyroelectric Nanogenerators

**Figure 2:**
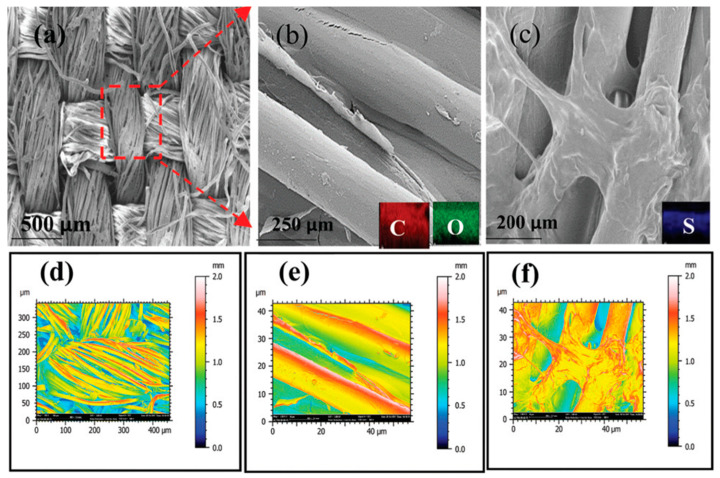
Reduced graphene oxide poly(3,4-ethylenedioxythiophene): poly (styrenesulfonate) (rGO-PEDOT:PSS) film-coated fabric of the flexible and washable thermoelectric nanogenerator fabricated by Khoso et al. [78]. This nanogenerator has potential application for harvesting green energy from human body heat. FESEM images with magnifications of (**a**) 500 μm and (**b**) 250 μm rGO-coated fabric and (**c**) 200 μm of rGO-PEDPT:PSS coated fabric. (**d**–**f**) Color mapping of SEM images’ infrared rendering. Reprinted with permission from [78]. Copyright ©2021, Royal Society of Chemistry.

Another sort of green energy that may be obtained from the environment is thermal energy. This energy may be transformed into electric energy and used to power low-power electronic devices employing thermoelectric nanogenerators (TEGs) [76]. TEGs produce electricity by using the Seebeck effect to scavenge thermal energy caused by temperature differences between two thermoelectric (TE) materials (Figure 2). This temperature differential causes charge carriers to migrate from a high-temperature TE material to a low-temperature TE material [77,78]. A TEG’s voltage output is proportional to the temperature gradient. TEGs, on the other hand, need significant temperature gradients across TE materials. TEGs are classified into two types: rigid thermoelectric nanogenerators and flexible thermoelectric nanogenerators, with the latter depending on their deformation properties. Stretchable, compressible, collapsible, lightweight, tiny in volume, affordable, and simple are advantages of TEGs [79,80,81]. Flexible TEGs have the potential to be employed in waste heat recovery [82,83,84], portable electronics [85,86,87], and human health monitoring due to their properties [88,89,90].

Pyroelectric nanogenerators (PyENGs) use the variation in spontaneous polarization inside pyroelectric materials to transform heat energy into electric energy. This is generated by oscillations of electric dipoles caused by a change in time-dependent temperature [91,92]. The creation of electric current through materials having a non-center symmetrical crystalline structure when subjected to a time-dependent temperature gradient is referred to as the pyroelectric effect [93,94].

Pyroelectric nanogenerators have been identified as the energy collectors of the future, with the potential to be a viable energy technology for scavenging thermal energy in everyday life [94]. Thus, PyENGs and TEGs may have significant uses in powering future intelligent electronic sensors and IoT-connected wearable devices. More investigations on inorganic and organic materials, structure, performance, and reliability are required for the development of these nanogenerators.

### 2.3. Hybrid Nanogenerators

**Figure 3:**
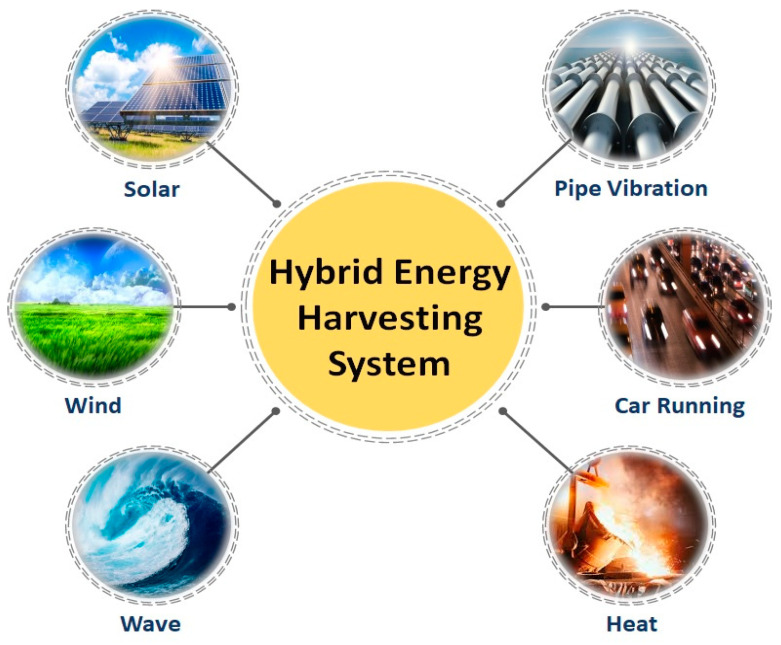
Potential applications of hybrid nanogenerators.

In the meantime, hybrid nanogenerators may harvest/recycle green energy from the environment by using several energy acquisition mechanisms or numerous connected nanogenerators with the same energy acquisition method (Figure 3). In hybrid nanogenerators, for example, piezoelectric, pyroelectric, triboelectric, and electromagnetic phenomena can be used. In comparison to individual nanogenerators, this nanogenerator type can provide high and efficient power density [95]. Recent research has led to the development of hybrid nanogenerators based on piezoelectric–pyroelectric [96,97,98], triboelectric–piezoelectric [31,99,100,101,102,103,104,105,106,107,108,109,110,111,112,113,114,115,116,117], electromagnetic–triboelectric [118,119,120,121,122,123,124,125,126,127,128,129,130,131,132], triboelectric–piezoelectric–pyroelectric [133,134,135,136], triboelectric–piezoelectric–electromagnetic [137,138,139,140,141,142,143,144,145,146,147,148], and photovoltaic–triboelectric effect [149,150,151,152,153,154].

## 3. Performance and Applications

**Figure 4:**
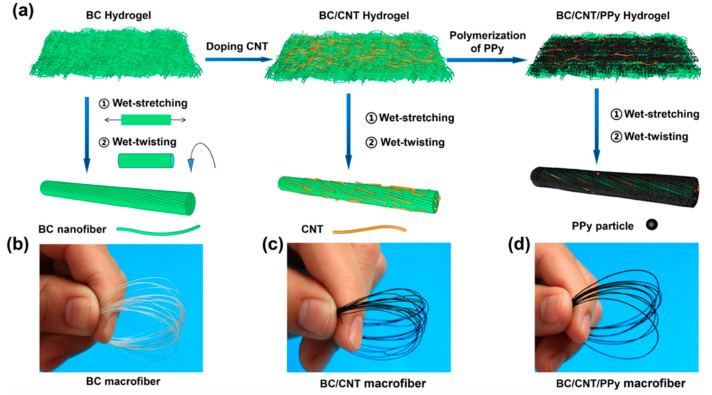
(**a**) Schematic view of the fabrication process of BC, BC/CNT/PPy macrofibers used in the fabric-based TENG developed by Hu et al. [155]. Images of (**b**) BC macrofibers, (**c**) BC/CNT macrofibers, and (**d**) BC/CNT/PPy macrofibers. Reprinted with permission from [155]. Copyright ©2022, Springer Nature.

**Figure 5:**
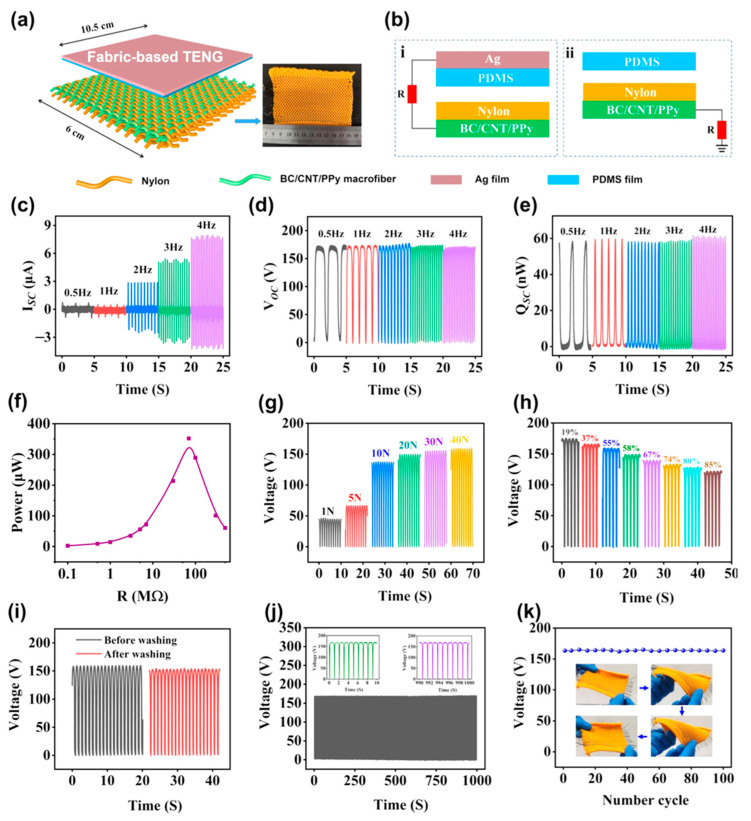
(**a**) Schematic view of the main components and materials of the fabric-based TENG designed by Hu et al. [155]; (**b**) two operating modes of the fabric-based TENG, (**i**) contact-separation mode and (**ii**) single electrode mode. Response of the (**c**) short-circuit current, (**d**) open-circuit voltage, and (**e**) transferred charges of the fabric-based TENG under different frequencies. (**f**) Results of the instantaneous power in relation to external load resistance, measurements of the output voltage of the fabric-based TENG considering (**g**) several impact forces at 1 Hz, (**h**) relative humidity variations, (**i**) before and after washing, (**j**) contact-separation mode with a frequency of 1 Hz during 100 s, and (**k**) mechanical strains with repetition of 100 cycles. Reprinted with permission from [155]. Copyright ©2022, Springer Nature.

**Figure 6:**
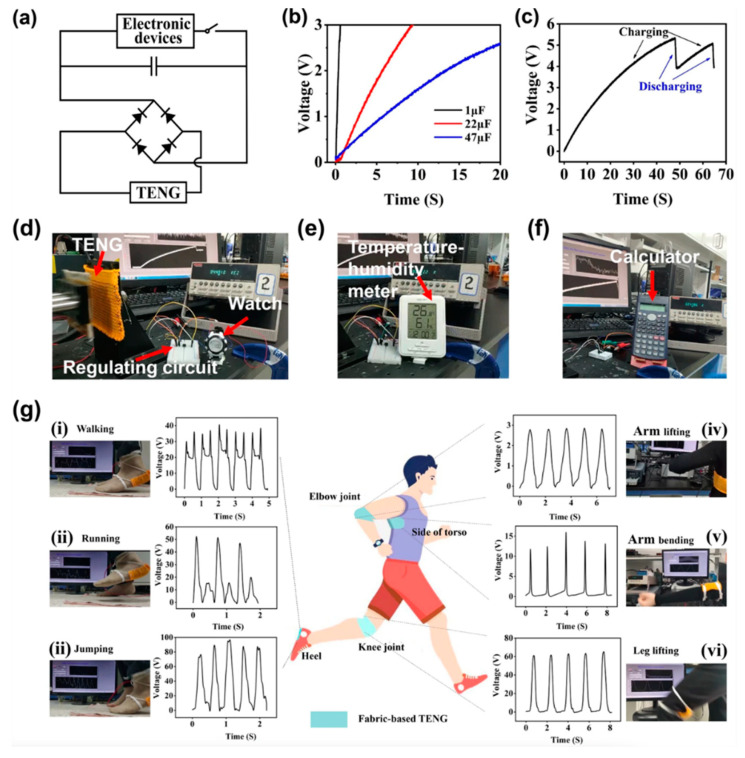
Applications of the fabric-based TENG were reported by Hu et al. [155]. (**a**) Diagram of the fabric-based TENG with rectifier bridge circuit for charging capacitors and powering electronic devices. (**b**) Response of the charging process of three commercial capacitors when the TENG is working in contact-separation mode with a frequency of 1 Hz. (**c**) Real-time measurements of the capacitor voltage, which is used for powering an electronic watch. (**d**) An electronic watch, (**e**) a temperature–humidity meter, and (**f**) a calculator powered using the fabric-based TENG with capacitors of 22 μF, 47 μF, and 100 μF, respectively. (**g**) Photographs and output voltages of the fabric-based TENG working as a self-powered device fixed to different sections of the human body for monitoring the body motion, (**i**) walking, (**ii**) running, (**iii**) jumping, (**iv**) arm lifting, (**v**) arm bending, and (**vi**) leg lifting. Reprinted with permission from [155]. Copyright ©2022, Springer Nature.

Hu et al. [155] designed an eco-friendly fabric-based TENG for converting biomechanical energy into electric energy, which can then be utilized to drive self-powered gadgets and wearable electronic sensors. This energy may be acquired by everyday human movements including leaping, jogging, walking, arms lifting, arms bending, and leg lifting. This TENG is made up of cellulose-based conductive macrofibers with key properties such as being super-strong, biodegradable, and washable. As illustrated in Figure 4, these microfibers were created by wet-stretching and wet-twisting bacterial cellulose (BC) hydro-gel with polypyrrole (PPy) and carbon nanotubes (CNTs). The microfibers were woven into a nylon fabric to generate the cellulose-based/nylon macrofiber. In this scenario, nylon serves as a positive triboelectric material, and a silver thin membrane is attached to a PDMS thin membrane to form a PDMS/silver film. As a result, the TENG features a cellulose-based/nylon macrofiber fabric that acts as a friction layer/electrode and a PDMS/silver layer that acts as a second friction film/electrode. The proposed microfibers demonstrated great tensile strength (449 MPa), strong electrical conductivity (5.32 Scm^−1^), and good stability. The highest open-circuit voltage of the TENG is 170 V, the short-circuit current is 0.8 µA, and the output power is 352 µW. (Figure 5). Furthermore, these TENG may function as self-powered devices for tracking human body motions (Figure 6).

**Figure 7:**
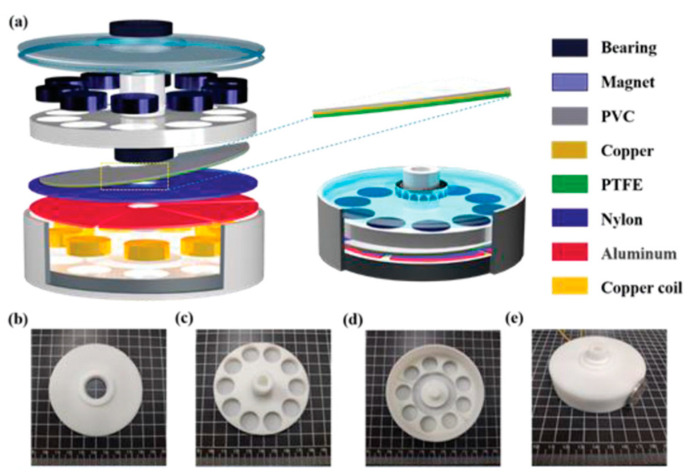
The TEHG structure developed by Zhao et al. [156]. (**a**) Schematic view of the main components and materials of the TEHG. (**b**) Image of the sealing cover of the cylindrical shell. (**c**) Image of the rotor disk. (**d**) Image of the cylindrical shell. (**e**) Image of the assembled structure of the TEHG. Reprinted with permission from [156]. Copyright ©2021, John Wiley and Sons.

**Figure 8:**
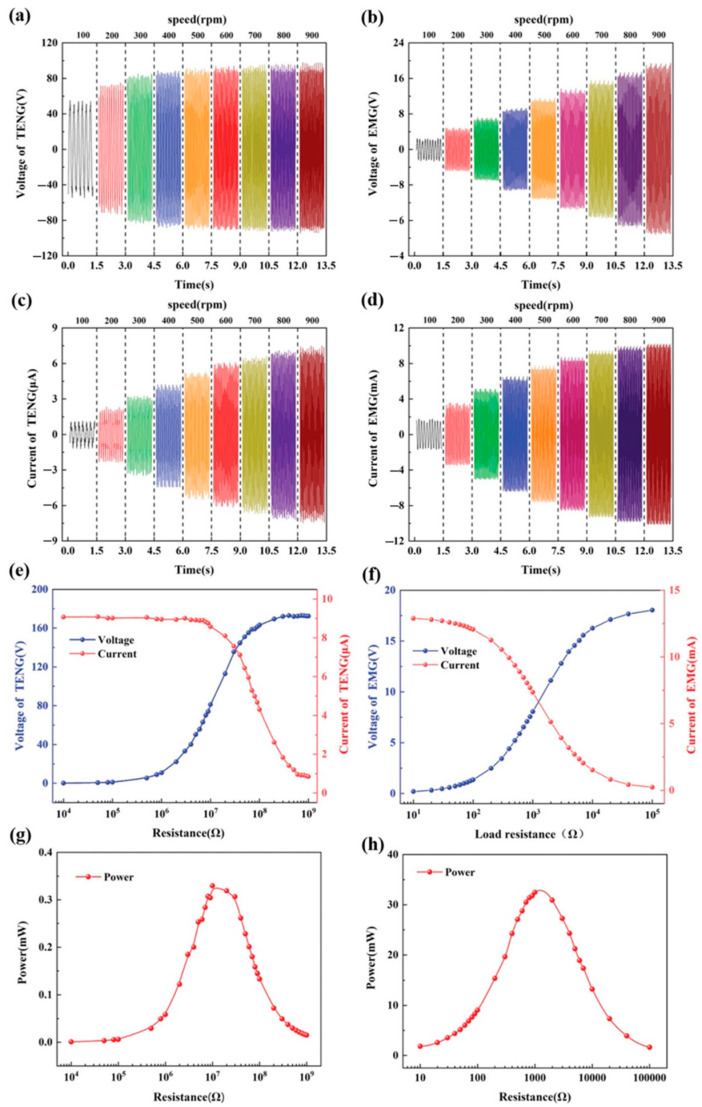
The output response of the TEHG was reported by Zhao et al. [156]. (**a**,**b**) The output open-circuit voltage of the TENG and EMG under several rotation speeds. (**c**,**d**) The output short-circuits current under several rotation speeds. (**e**,**f**) The output voltages and currents of the TENG and EMG as a function of external load resistance at a rotation speed of 400 rm. (**g**,**h**) The average output power of the TENG and EMG as a function of external load resistance at a rotation speed of 400 rpm. Reprinted with permission from [156]. Copyright ©2021, John Wiley and Sons.

**Figure 9:**
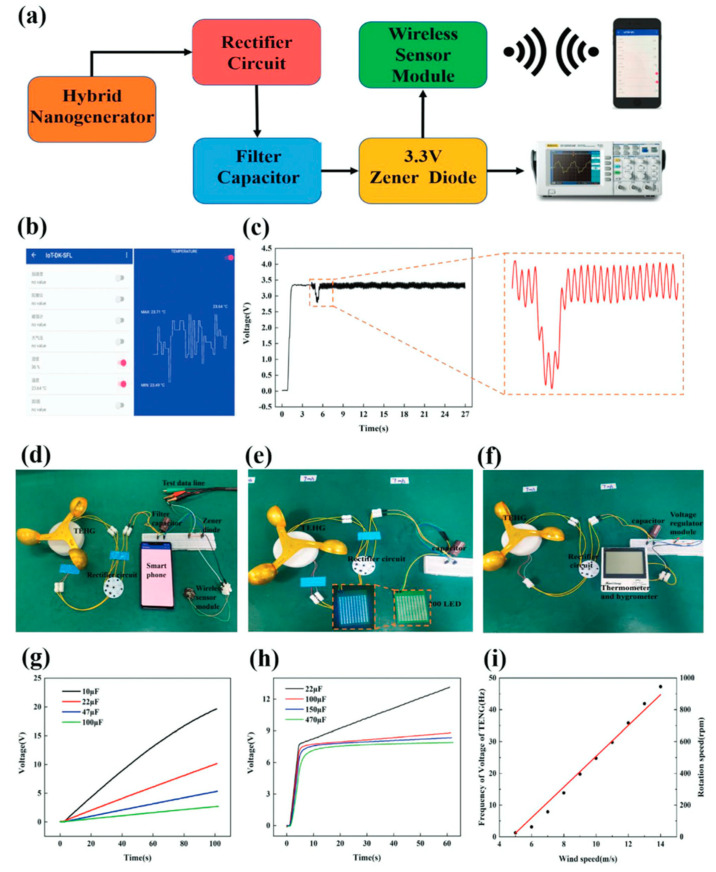
The application of the TEHG was proposed by Zhao et al. [156]. (**a**) Schematic view of the designed circuit for continuously supplying energy for the wireless sensor network node. (**b**) Mobile phone display to receive sensor data. (**c**) Mobile phone display to receive voltage data. (**d**) The test circuit of the TEHG to power the wireless sensor network node. (**e**) TEHG used to light up 200 LEDs. (**f**) TEHG supplies power to the thermometer and hygrometer device. (**g**) TENG is used to charge different capacitors. (**h**) TEHG is employed to charge several capacitors. (**i**) Relationship between the output voltage frequency, wind speed, and rotation speed. Reprinted with permission from [156]. Copyright ©2021, John Wiley and Sons.

Zhao et al. [156] produced a triboelectric–electromagnetic hybrid nanogenerator (TEHG) that can gather wind energy while also powering electronic gadgets. This nanogenerator is made up of a TENG that operates in the sliding independent triboelectric-layer mode and an EMG that operates in the rotating mode. Figure 7 depicts the structure and materials of the TEHG, which is made up of a rotor and a stator. The stator has a cylindrical shell that is sealed, while the rotor has a disk and a projecting cylinder. This cylinder features an inside cylinder that can accommodate wind cups to convert environmental wind energy into mechanical energy. The cylindrical magnets of the EMG are positioned in ten cylindrical grooves on the upper surface of the rotor disk. The completed TEHG structure has an outside diameter of 80 mm and a height of 20 mm. The TENG employs PTFE and nylon as triboelectric layers that are in touch with one another. The PTFE functions as a 0.3 mm thick negative friction layer, while the nylon acts as a positive friction substance. Six aluminum electrodes are joined as interdigital electrodes on the nylon layer. Furthermore, the bottom of the shell has nine grooves for installing the copper coils of the EMG. These coils are wired in series to boost the output signal. The rotation of the TEHG structure caused by the wind source causes surface charge transfer between the two triboelectric layers. During the TEHG rotation process, an alternating current with a changing direction is produced. Figure 8 displays the TEHG’s output open-circuit voltage and short-circuit current readings at various rotation speeds. Peak-to-peak voltage and peak-to-peak current of the TENG grow from 106 V to 190 V and 2.27 µA to 14.6 µA, respectively, with rotation speeds ranging from 100 rpm to 900 rpm (14 m/s of wind speed). The output response of the EMG is determined by the relative rotation of the magnet and coil based on electromagnetic induction (Faraday’s law). The peak-to-peak voltage and peak-to-peak current rise from 5 V to 38 V and 3.3 mA to 20 mA, respectively, as the rotation speed increases from 100 rpm (5 m/s of wind speed) to 900 rpm. The TENG has a maximum average output power of 0.33 mW at an ideal load resistance of 12 MΩ. The EMG, on the other hand, has a maximum average output power of 32.87 mW and a maximum load resistance of 1.25 KΩ. The TEHG was evaluated for its ability to provide energy to wireless sensor network nodes. For this, Zhao et al. designed a circuit that incorporates a test device for measuring voltage changes and a cell phone for receiving data from the node (Figure 9). The TEHG was used to light up 200 LEDs in tandem and power an ambient humidity and temperature sensor at a rotation speed of 400 rpm (9 m/s of wind speed).

**Figure 10:**
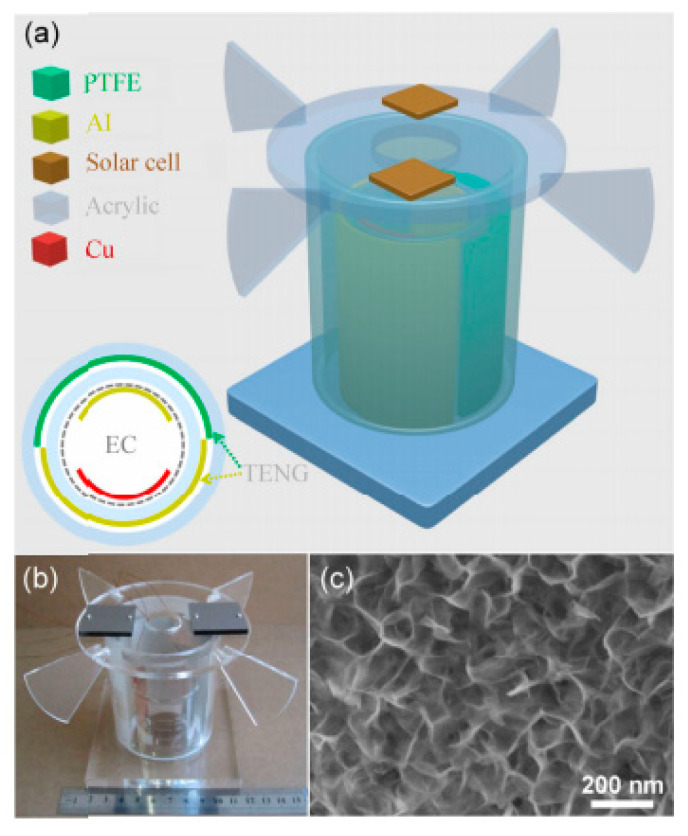
(**a**) Schematic view of the main components of the hybrid energy cell developed by Wu et al. [157]. (**b**) Image of the fabricated hybrid energy cell that includes solar cells and electrochemical cells placed on and in the TENG, respectively. (**c**) SEM image of Al film surface of the TENG, which was modified using nanostructures. Reprinted with permission from [157]. Copyright ©2014, Tsinghua University Press and Springer-Verlag Berlin Heidelberg.

**Figure 11:**
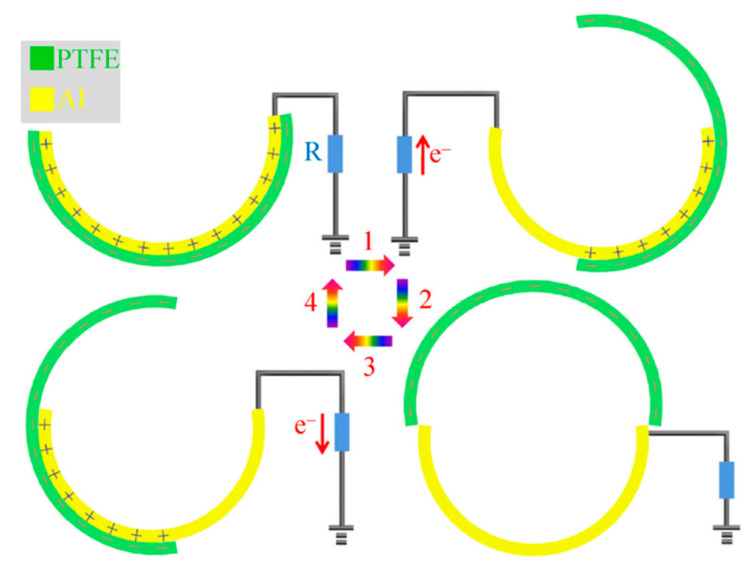
Stages of the operation principle of the TENG used in the hybrid energy cell fabricated by Wu et al. [157]. Reprinted with permission from [157]. Copyright ©2014, Tsinghua University Press and Springer-Verlag Berlin Heidelberg.

**Figure 12:**
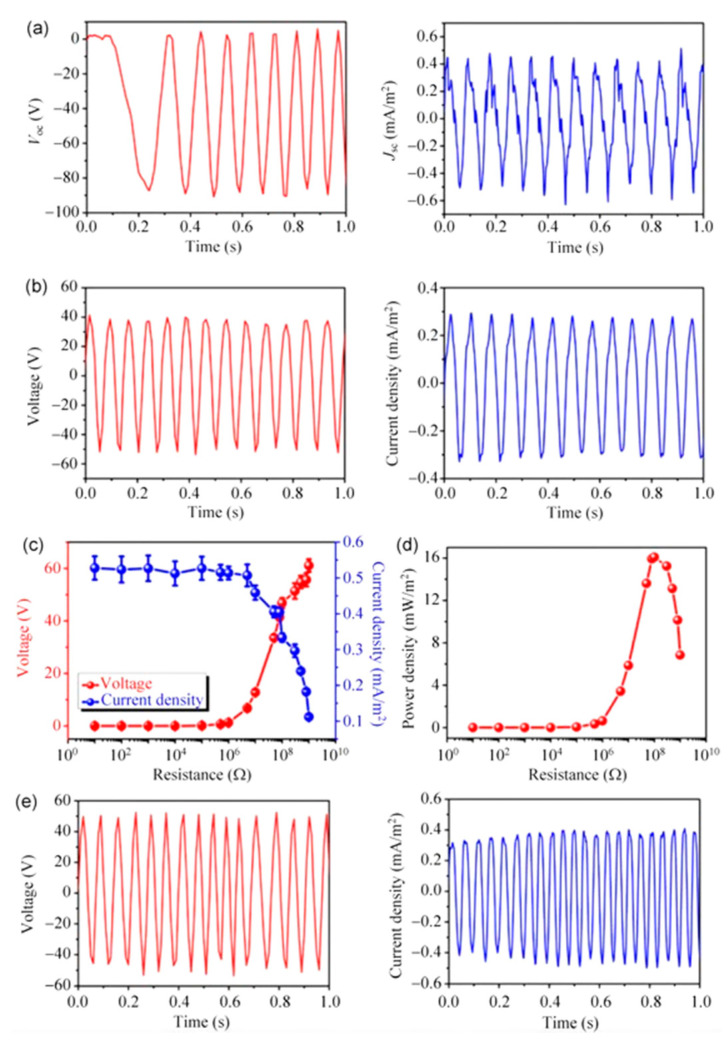
(**a**) Experimental results of the open-circuit voltage and short-circuit current density of the TENG using one strip unit. (**b**) Output voltage and current density of the TENG with one strip unit considering a load resistance of 100 MΩ. (**c**) Variation in the output voltage and current density of the TENG as a function of load resistance. (**d**) Response of the power density as a function of load resistance. (**e**) Output voltage and current density of the TENG with two strip units. Reprinted with permission from [157]. Copyright ©2014, Tsinghua University Press and Springer-Verlag Berlin Heidelberg.

Wu et al. [157] introduced a hybrid energy cell (Figure 10) that combines a TENG, an electrochemical cell (EC), and eight amorphous silicon-based solar cells (SCs) to gather wind, chemical, and solar energies from the environment simultaneously or independently. This hybrid energy cell might power low-power electronic devices such as wind speed sensors and temperature sensors. The key benefit of this technology is its capacity to scavenge three separate energy sources at the same time, which improves the usage of energy from the environment. A polytetrafluoroethylene (PTFE) film and an Al film are bonded to two acrylic tubes to form the TENG. The periodic contact/separation between the PTFE film and the Al film can generate charge transfer between the Al electrode and the ground by utilizing the coupling between the triboelectric effect and the electrostatic effect (Figure 11). First, both the Al and PTFE films are in an aligned position, where the two surfaces are completely in touch with one other. The two films have opposing charge polarities and are entirely balanced in this configuration, resulting in no electron flow across from Al film to PTFE film. This sequence is completed, and the mismatch between the two films is obtained. Since the relative rotation of both films continues, the PTFE film travels back to touch the Al film, creating electrons that flow from the Al electrode to the ground. This electrostatic induction action can lead the TENG’s output signals to increase, indicating that the charges are entirely balanced. An alternating electric shape output is obtained during a TENG operation cycle. Figure 12 depicts the manufactured TENG’s output signals. This TENG has an open-circuit voltage approaching 90 V, a short-circuit current density close to 0.5 mA/m^2^, and a maximum power density of 16 mW/m^2^, allowing it to power up 20 blue light-emitting diodes directly (LEDs). For charging a capacitor, the hybrid energy cell outperformed separate energy units significantly. The hybrid device’s gathered energy can be stored in a Li-ion battery as a controlled power module for powering electronic equipment. Increasing the surface roughness and effective surface area of the triboelectric material induces a higher triboelectric charge density and improves TENG output performance.

The collagen fibrils of vegetables, fruits, and plants may be the responsible constituents of these natural materials’ piezoelectricity. The piezoelectricity in collagen fibrils is caused by intermolecular hydrogen bonding, which results in a uniaxial orientation of the molecular dipoles [158]. Tomato peels (TPs), for example, include 16 different amino acids [159] and non-centrosymmetric properties due to their low symmetrical orthorhombic and mono-clinic space groups, which might contribute to the piezoelectric effect [159]. Furthermore, the TPs feature structures with significant porosity, which causes additional displacement owing to applied external stresses, boosting the TPs’ piezoelectricity [160,161]. Furthermore, the hydroxyl groups in TPs’ lutein and zeaxanthin contribute to their piezoelectricity. Hydrogen bonding occurs in the hydroxyl group due to the extremely electropositive hydrogen and electronegative oxygen atoms [160]. According to the findings of these studies, TPs can be employed to generate piezoelectric energy.

**Figure 13:**
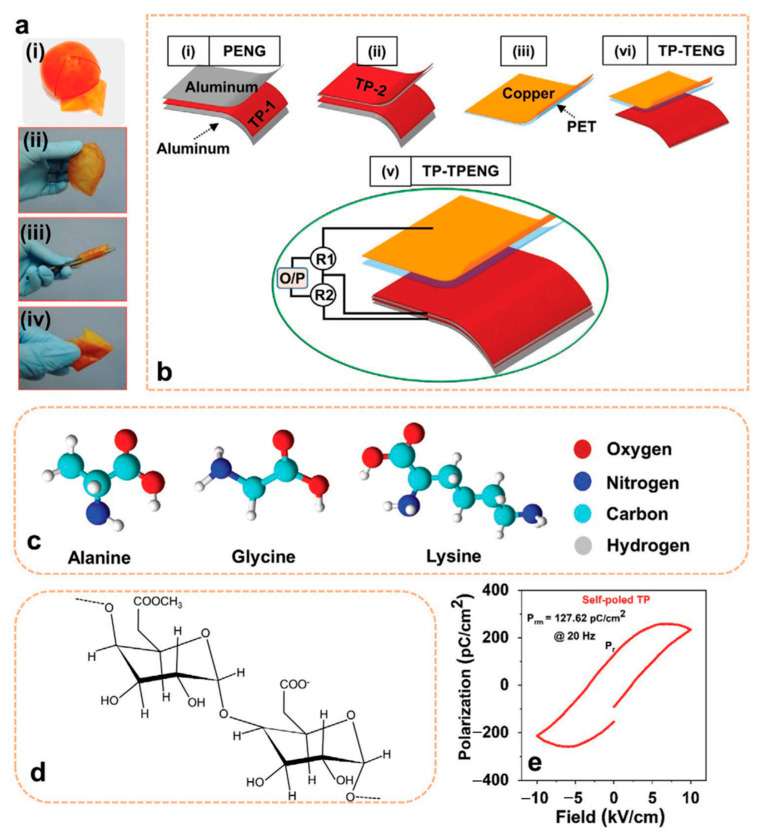
(**a**) Images of (**i**) peeling of a tomato, (**ii**) unfolded TP, (**iii**) folded TP, and (**iv**) rolled TP. (**b**) Schematic view of the main elements and materials used in the hybrid nanogenerator. (**c**) Structures of the three amino acids (alanine, glycine, and lysine) of the TP that allow the presence of C, O, N, and H. (**d**) Schematic view of the carbonyl and hydroxyl groups in the chain of the TP pectin structure. (**e**) Response of TP ferroelectric hysteresis considering a frequency of 20 Hz. Reprinted with permission from [160]. Copyright ©2021, John Wiley and Sons.

**Figure 14:**
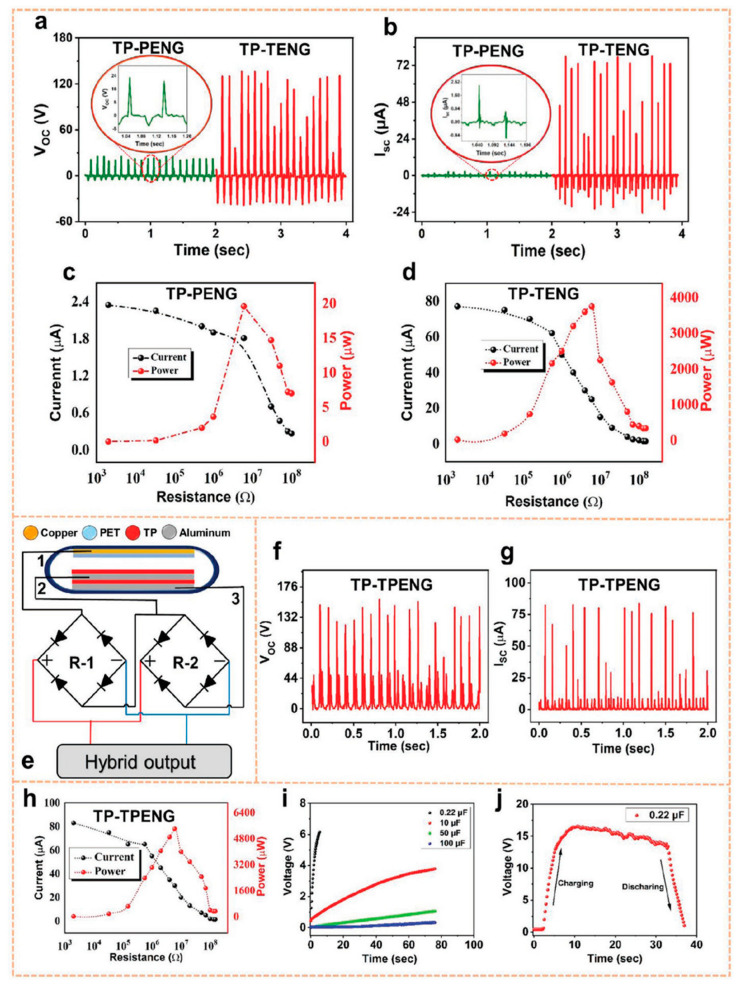
Electric output performance of the TP-PENG, TP-TENG, and TP-TPENG fabricated by Saqib et al. [160]. (**a**) Open circuit voltage and (**b**) short circuit current of the TP-based nanogenerator without considering the combination of both piezo and triboelectric effects. Output current and instantaneous power of the (**c**) TP-PENG and (**d**) TP-TENG as a function of the load resistance. (**e**) Schematic view of the main elements and materials of the hybrid nanogenerator. (**f**) Open circuit voltage and (**g**) short circuit current of the TP-TPENG. (**h**) Variation in the generated output current and instantaneous power of the TP-TPENG related with several external load resistances. (**i**) Charging curve of four different capacitors employing TP-TPENG. (**j**) The charging and discharging behavior of capacitor using hybrid nanogenerator. Reprinted with permission from [160]. Copyright ©2021, John Wiley and Sons.

**Figure 15:**
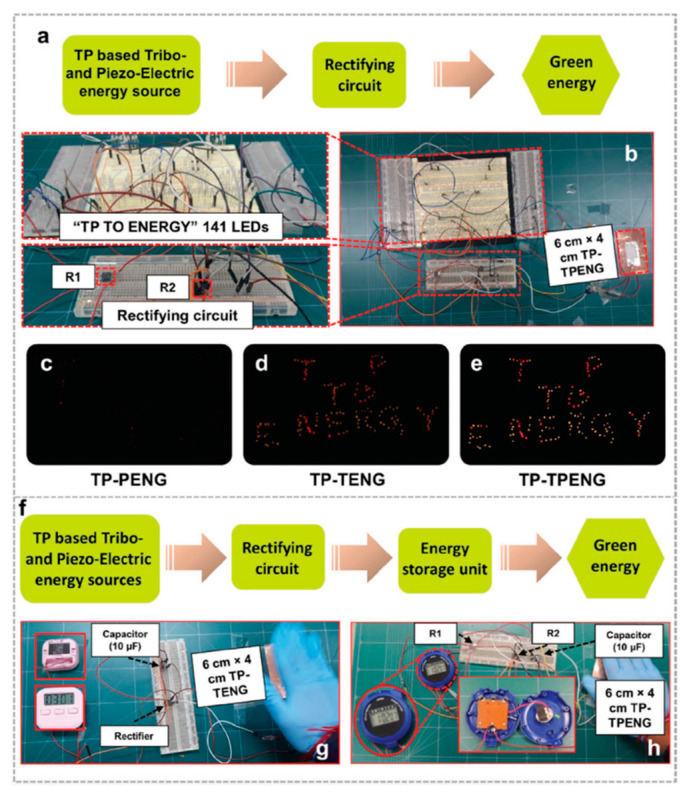
Application of the TP-based nanogenerators reported by Saqib et al. [160]. (**a**) Schematic diagram and (**b**) application of the TP-TPENG with rectifier circuit to light up LEDs. Lighted LEDs using (**c**) TP-PENG, (**d**) TP-TENG, and (**e**) TP-TPENG, respectively, under a simple hand pressing force. (**f**) Schematic diagram of the TP-TPENG with rectifier circuit and energy storage unit. Different stopwatches are powered using (**g**) TP-TENG and (**h**) TP-TPENG, respectively. Reprinted with permission from [160]. Copyright ©2021, John Wiley and Sons.

Saqib et al. [160] examined the triboelectric and piezoelectric action of tomato peel (TP) in order to build a hybrid nanogenerator (TP-TPENG) with bio-organic nature materials for collecting green energy with potential applications in pollution-free and self-powered devices. The tomato’s very porous structure boosts the TP-output TPENG’s responsiveness. The open-circuit voltage, short-circuit current, and highest instantaneous power of a TP-based piezoelectric nanogenerator (TP-PENG) are 24.5 V, 2.5 µA, and 19.5 µW, respectively. The open-circuit voltage, short-circuit current, and highest instantaneous power of the TP-based triboelectric nanogenerator (TP-TENG), on the other hand, are 135 V, 81 µA, and 3750 µW, respectively. The combination of triboelectric and piezoelectric effects resulted in an enhanced TP-TPENG output response with a rectifier circuit. The rectified open circuit voltage, short circuit current, and maximum instantaneous power of this TP-TPENG are 150 V, 84 µA, and 5400 µW, respectively. Thus, TPs may be used to create unique non-toxic and eco-friendly hybrid nanogenerators based on their piezoelectric and triboelectric capabilities. This hybrid nanogenerator powered 141 commercial LEDs while also charging a 10 µF capacitor. Figure 13 depicts the primary components and materials of the hybrid nanogenerator. The TPs’ hydroxyl and carbonyl groups contribute to their piezoelectric and triboelectric characteristics. Furthermore, TPs offer high flexibility and robustness. Figure 14 depicts the electrical output behavior of the TP-based nanogenerator when the piezoelectric and triboelectric effects are taken into account. The electrical output performance of the TP-TENG is superior to that of the TP-PENG. On the other hand, the combined responsiveness of TP’s piezoelectric and triboelectric capabilities allows for a TP-TPENG with more superior electric output response than both TP-PENG and TP-TENG. The alternating output signal of the hybrid nanogenerator is rectified using two rectifier circuits. In addition, the hybrid nanogenerator was used to charge four different capacitors (0.22 µF, 10 µF, 50 µF, and 100 µF). The three nanogenerators were used to power several commercial LEDs. In addition, TP-TENG and TP-TPENG with rectifier circuits were used to power many commercial stopwatches (Figure 15).

**Figure 16:**
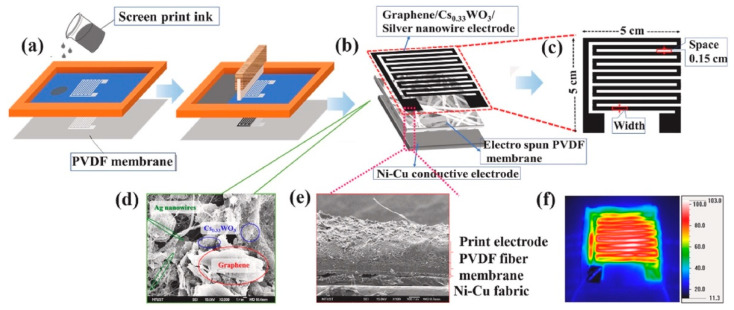
The main components and materials of the PyNG were reported by Gokana et al. [91]. (**a**) Schematic diagram of the elaboration of the serpentine electrode (SRE) using screen-printing. (**b**) Schematic diagram of structural design and materials of the SRE PyNG. (**c**) Dimensions of the SRE pattern. FESEM images of (**d**) surface and (**e**) cross-sectional view of the PyNG. (**f**) Temperature response of the PyNG using IR thermographic. Reprinted with permission from [91]. Copyright ©2022, Elsevier B.V.

**Figure 17:**
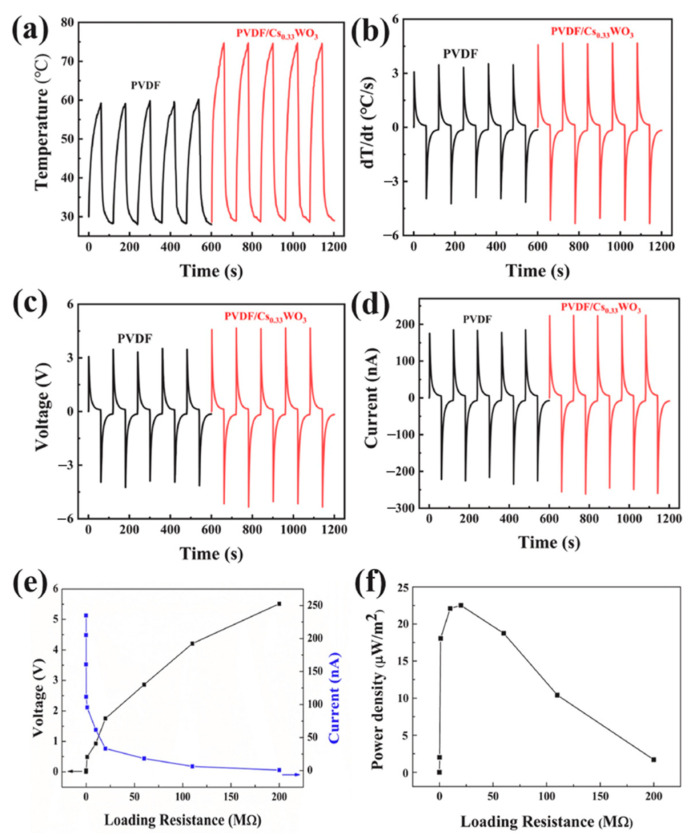
The thermal and electrical output performance of the PyNG developed by Gokana et al. [91]. (**a**) Temperature variation, (**b**) rate of temperature shift, (**c**) output voltage, and (**d**) output current of both pure PVDF PyNG and PVDF/Cs_0.33_WO_3_ PyNG. (**e**) Output voltage and current of PVDF/Cs_0.33_WO_3_ PyNG as a function of load resistance. (**f**) Output power density of PVDF/Cs_0.33_WO_3_ PyNG as a function of load resistance. Reprinted with permission from [91]. Copyright ©2022, Elsevier B.V.

**Figure 18:**
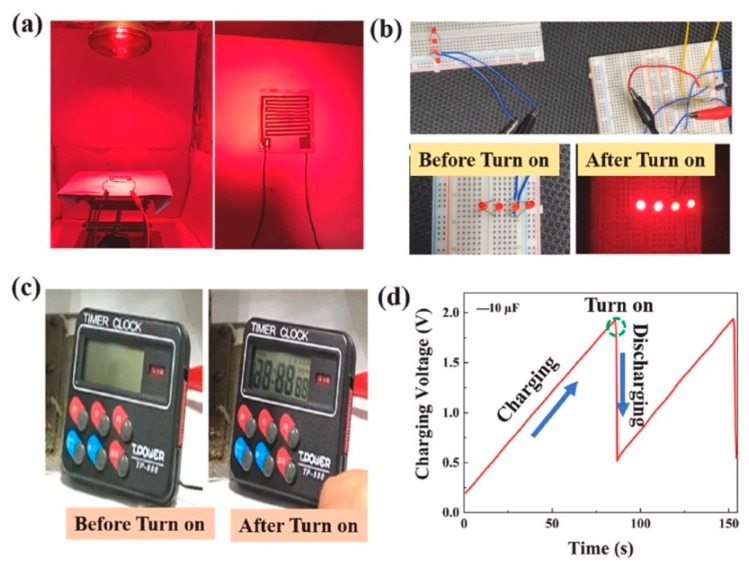
Application of the PVDF/Cs_0.33_WO_3_ PyNG designed by Gokana et al. [91]. (**a**) Experimental setup of the PyNG to turn on (**b**) four lighted LEDs and (**c**) display LCD. (**d**) Charging and discharging voltage of 10 μF capacitor using the PVDF/Cs_0.33_WO_3_ PyNG. Reprinted with permission from [91]. Copyright ©2022, Elsevier B.V.

Gokana et al. [91] developed a pyroelectric nanogenerator (PyNG) capable of producing electric energy from waste heat in the environment. As illustrated in Figure 16, this PyNG is made using a screen-printed serpentine electrode (SRE) that has been modified with cesium tungsten bronze (Cs_0.33_WO_3_). Furthermore, Cs_0.33_WO_3_ was applied to both the electrode and the PVDF sheets. With a load resistance of 20 ΩM, the PyNG with 7 wt% Cs_0.33_WO_3_ can reach a temperature of 121 °C and electrical output voltage, current, and power density of 4.36 V, 214 nA, and 23.38 μW/m^2^, respectively. A liquid crystal display (LCD) and four LEDs were powered by the proposed PyNG. This PyNG is an alternate source for capturing solar energy and powering low-power electrical gadgets. Figure 16 depicts the various materials and components utilized in the fabrication of the PyNG, as well as the temperature values of its electrodes determined by IR thermographic imaging. The thermoelectric conversion behavior of the PyNG was evaluated by measuring its thermal and electrical output responses during heating and cooling at 8 mHz switching frequency. Under near-infrared (NIR) radiation, the PVDF PyNG with 7 wt% Cs_0.33_WO_3_ quickly registered 75 °C and reverted to 29 °C after 60 s of radiation removal. Using the same experimental conditions, PVDF PyNG without Cs_0.33_WO_3_ reached 58 °C and reverted to 29 °C. As a result, the rate of temperature changes of PVDF/Cs_0.33_WO_3_ PyNG was increased by over 27% when compared to pure PVDF PyNG. Furthermore, as compared to the pure PVDF PyNG, the electrical output voltage and current of the PVDF/ Cs_0.33_WO_3_ PyNG increased by around 26% and 16%, respectively (Figure 17). The PyNG’s better performance might be attributed to its high photothermal conversion behavior and NIR light absorption. When NIR light is shone on the improved PyNG with a 10 µF capacitor, the PyNG may activate four LEDs and display an LCD, as illustrated in Figure 18.

Table 1 reported the comparison of the main characteristics of various nanogenerator types, including the transduction mechanism, energy source, materials, advantages, and weaknesses. For a single transduction mechanism, the triboelectric nanogenerators have important characteristics such as high electrical output performance, good stability, simple structure, and cost-efficient fabrication. In addition, this nanogenerator can be designed to convert different energy sources (e.g., biomechanical, water waves, environmental vibrations, and wind) into electrical energy. On the other hand, the hybrid nanogenerators present an enhanced electrical output performance in comparison with nanogenerators based on a single transduction mechanism. The hybrid nanogenerators that include the triboelectric effect have the best operation parameters such as compact and simple structure, good flexibility, stability, low-cost manufacturing, and high electrical output performance.

## 4. Challenges and Perspectives

This section discusses the main difficulties and prospects for nanogenerators in terms of design, materials, output performance, reliability, and prospective applications.

### 4.1. Design

The nanogenerator’s design phase is critical to achieving the greatest performance for certain applications. This stage of design must take into account the various requirements and working circumstances of the prospective application of the nanogenerators. Thus, nanogenerator designers should investigate the required electric power, size and weight limitations, working time, and environmental conditions (relative humidity, temperature, pressure, radiation, wind, vibrations, dust, and so on), green energy acquisition mechanisms, materials more suitable for nanogenerator electromechanical behavior, fabrication process, packaging type, minimum electronic components, and so on. Analytical and numerical modeling may be utilized in the design of nanogenerators to find the best operation principle, materials, and electromechanical configuration that allow for the safety and reliability of the nanogenerators under various working conditions.

Furthermore, numerical simulation tools such as ANSYS, COMSOL, NASTRAN, ABAQUS, and MATLAB may be used to evaluate the performance of nanogenerators. As a result, the designers may create bidimensional or tridimensional models of the nanogenerators, complete with their major components, materials, and operating conditions. However, due to errors in the selection of the materials’ characteristics, load values and analysis types, boundary conditions, and mesh size, the findings of the analytical and numerical simulation models of the nanogenerators might have a large error percentage in comparison to experimental results. To reduce this error percentage, designers should evaluate the important elements that determine the performance of the nanogenerators, such as genuine values of material characteristics, the right selection of load and boundary conditions, and the mesh quality and type of the suggested models.

### 4.2. Materials

The appropriate material selection for nanogenerators is critical for improving their performance and reliability for each prospective application. Each nanogenerator application necessitates unique performance characteristics in order to maximize the green energy collecting process from various natural sources and under varying climatic conditions. Thus, the design of a specific nanogenerator requires superior materials to meet the electrical signal requirements and performance stability for future applications. Nanogenerators for biomechanical applications, for example, may need stretchable, lightweight, and flexible materials to improve their output electrical responsiveness and mechanical behavior [162]. These materials must have the structural strength to minimize wear and mechanical failures in this application, as well as low density to reduce the weight of the nanogenerators. Furthermore, the operation of nanogenerators might be harmed by environmental dampness. The application of hydrophobic materials or materials to nanogenerator packing can be used to solve the humidity problem. For instance, nanogenerators for blue energy harvesting are built with specific packaging made of low-density materials that are resistant to corrosion and solar radiation. Nanogenerators that operate under mechanical vibrations, on the other hand, should be designed with materials that have adequate structural strength to reduce structural failures caused by cracks, wear, fatigue, or fracture. Recent nanogenerator research [39,63,64,160,163,164,165,166,167,168,169] has focused on organic or waste materials from the environment, such as tomato, chitin, eggshell, fish swim bladder, spider silk, peanut shell, sunflower husks, rice paper, garbage soda cans, silk fibroin, coconut husk, and so on. However, measuring the piezoelectric and triboelectric properties of these materials is extremely difficult. Another significant constraint is the analytical modeling of the piezoelectric and triboelectric response of organic materials. More research is needed to determine the piezoelectric and triboelectric effects of organic or waste materials employed in nanogenerators for green energy harvesting.

### 4.3. Energy Storage and Electrical Interfaces

The electrical output performance of nanogenerators can be impacted by changes in ambient circumstances and green energy stability, which can vary over time and exhibit erratic behavior. Due to these circumstances, the output electrical signals of nanogenerators might exhibit erratic behavior. In addition, most electronic equipment must be powered by DC voltage and current. The nanogenerators require rectifier circuits to convert their AC output electrical impulses into DC signals in order to power these devices. These DC signals must also be stored in capacitors or batteries in order to power electronic devices with controlled electrical signals. The development of effective energy storage devices is an intriguing research problem for nanogenerators.

Another important challenge of the nanogenerators is the development of electrical interfaces that achieve high efficiency with minimum power consumption [170,171,172]. For instance, these electrical interfaces could be self-powered and consider cold-start circuit architectures [173,174]. To reduce the size of the nanogenerators, the electrical interfaces should have a small footprint. It could be obtained using the Application-Specific Integrated Circuit (ASIC) implementation. Moreover, the electrical interfaces could be adaptive in order to maximize the harvested power, considering low-power maximum power point tracking (MPPT) algorithms [174,175,176,177].

### 4.4. Fabrication

To create nanogenerators for commercial uses, large-scale manufacture should be enabled. Alternatives to this difficulty include no-complex manufacturing procedures and new infrastructure with flexible phases for new nanogenerator designs and use. Furthermore, a low-cost production technique with few processing steps is critical for the market feasibility of nanogenerators. Another possibility to reduce nanogenerator manufacture costs is to re-use inorganic or organic materials from trash [63]. Furthermore, future nanogenerator manufacturing processes may contain biodegradable and environmentally benign materials.

### 4.5. Reliability

Future studies will focus on the stability and reliability of the electromechanical behavior of nanogenerators. For commercial applications, nanogenerators must provide output electrical signals that are stable throughout time. During the life of a nanogenerator, its electrical and structural components may have performance issues owing to abrasion, mechanical impact, crack development, fatigue, humidity, radiation, high temperature, environmental pollution, and other factors. To extend the life of nanogenerators, basic structural layouts with the fewest number of electrical and mechanical components should be considered. Electrical or mechanical failures of some of these components can affect the operation of nanogenerators with complicated structural designs that involve multiple components. To decrease the possibility of electrical and mechanical failures in the various nanogenerator components, the use of robust materials and appropriate packaging might increase the service life of nanogenerators.

## 5. Conclusions

The most recent advances in nanogenerators for green energy harvesting via various transduction processes were discussed. Triboelectric, piezoelectric, electromagnetic, and thermoelectric effects were all explored in these processes. The principles of operation and materials of several nanogenerators are reviewed. In addition, the behavior of the output electrical signals (voltage, current, and power) of multiple nanogenerators was considered, taking into account the combination of green energy acquisition processes. It was stated that nanogenerators were used to power several commercial electronic products. This review also discussed the problems and perspectives of nanogenerators in design, materials, energy storage, fabrication, and reliability.

## Figures and Tables

**Table 1:** Summary of the main characteristics of several nanogenerators used to harvest different green energy sources.

Transduction Mechanism	Energy Source and Main Materials	Advantages	Weaknesses	Potential Application	Reference
Piezoelectric	Biomechanical PVDF/Gly-MoS_2_ composite film	High electro-mechanical performance, large-area compliant, long-time output signal stability, and low-power manufacturing	Performance depends on the Gly-MoS_2_ nanosheet’s content	Self-powered sensory systems, biomedical monitoring, and wearable electronics	[37]
Piezoelectric	Biomechanical PDMS/PPy composite film	Low-cost fabrication and flexible and robust devices	Performance depends on the PPy content	Self-powered biocompatible electronic devices	[38]
Piezoelectric	Biomechanical PVDF/coconut husk powder (CHP) composite film	Biowaste materials, simple fabrication process, and good electromechanical stability	Performance power depends on the CHP content	Biomedical devices and sustainable sensors	[39]
Piezoelectric	Biomechanical PVDF film	Flexible materials and low-cost fabrication	Electromechanical behavior requires more tests	Self-powered blood pressure sensors and wearable biomedical devices	[42]
Piezoelectric	Biomechanical 3D PPy/PVDF-poly-hexafluoropropylene (PHFP) composite film	Flexible materials and good performance stability	Performance depends on the 3D PPy content	Flexible biomedical devices	[44]
Triboelectric	Water waves and wind Cu electrodes, deionized water, and fluorinated ethylene–propylene tube	High output power density, simple structure, and easy fabrication process	Output power is highly dependent on the acceleration of water motion	Ship attitude sensors, multi-module devices, and ultra-sensitive sensor systems	[50]
Triboelectric	Water waves Stainless steel electrodes and PTFE film	High surface charge density, high output power, and low friction-induced loss	Wear of film by friction	Self-powered marine sensors, ocean buoys, and self-powered distributed energy for the marine IoT	[51]
Triboelectric	Water waves Cu electrodes and PTFE balls	Compact structure, ease of integration, and simple operation	Output performance depends on the wave direction and amplitude	Ocean buoys and cost-efficient beacon in night time marine operations	[52]
Triboelectric	Water waves Spring steel sheet and PFTE film	High electrical output performance for any direction of movement	Complex structure	Self-powered smart marine sensors and distributed power systems in oceans	[55]
Triboelectric	Biomechanical, environmental vibration and wind Ag electrodes, commercial VHB 4905 and Chitin films	Biodegradable materials, simple and cost-efficient fabrication, and high output performance	Performance depends on the chitin concentration	Self-powered flexible sensors, health monitoring of subtle pressures, non-contact sensing, and human–machine interfaces	[64]
Triboelectric	Biomechanical Cu electrodes, flourinated ethylene propylene (FEP) film	Simple operation and easy fabrication process	Wear of film by friction	Flexible electronic devices for real-time monitoring of human physiological states	[67]
Triboelectric	Biomechanical Cu and Ni fabrics and PDMS with BaTiO_3_ nanoparticles	Stable electric behavior, ultra-flexibility, mechanical durability, and cyclic washing ability	Complex fabrication	Self-powered, wireless, and intelligent monitoring of human motions, portable power sources, and multifunctional human–machine interfaces	[71]
Thermoelectric	Human body heat rGO and PEDOT:PSS-coated textile fabric	Improved thermoelectric efficiency, high flexibility, breathable, washable, and bendable textile fabric	Complex manufacturing process and performance depends on the rGO concentration	Self-powered flexible devices and wearable e-textiles	[78]
Thermoelectric	Human body heat Cu, PET, Bi_2_(Te_1−x_Se_x_)_3_, and (Bi_x_Sb_1−x_)_2_Te_3_	Good flexibility and high output power density	Complex manufacturing process	Self-powered flexible and wearable sensors	[80]
Thermoelectric	Thermal ITO/PET, PEDOT:PSS, and MoS_2_/graphene composite	Ultra-flexible and shape-adaptive	Complex manufacturing process and performance depends on the MoS_2_/graphene content	Self-powered temperature sensors	[83]
Thermoelectric	Thermal and human body heat PEDOT:PSS/single-walled carbon nanotube (SWCNT) composite fibers	High flexibility and good bending durability	Complex manufacturing process and performance depends on the SWCNT content	Wearable electronic devices	[85]
Thermoelectric	Human body heat PDMS/boro nitride, n-Bi_2_Te_3_, and p-Sb_2_Te_3_ composite films	Portable and good flexibility	Complex manufacturing process	Self-powered wearable sensors for monitoring of human physiological signals and body motions	[90]
Pyroelectric	Near-infrared light PVDF/Cs_0.33_WO_3_ composite film	High output performance and high charge/discharge stability	Performance depends on the Cs_0.33_WO_3_ content	Implantable stimulator, high sensitivity sensors, and self-powered electronic devices	[91]
Pyroelectric	Thermal Au and ITO electrodes and ferroelectric antimony sulfoiodide (SbSI)-TiO_2_ composite film	Simple fabrication process	Performance depends on the size and concentration of the TiO_2_ nanoparticles	Pyroelectric sensors	[92]
Piezoelectric–pyroelectric	Biomechanical MWCNT doped PVDF nanofibers	High electrical output performance and high mechano-sensitivity	Complex manufacturing process and performance depends on the MWCNT content	Biomedical sensors integrated with IoT and remote care of infectious diseases	[96]
Piezoelectric–pyroelectric	Biomechanical Graphene oxide (GO), graphene (Gr), and halloysite (HNT) nanofillers and PVDF nanofibers	Improved electrical output performance and thermal stability	Complex manufacturing process and performance depends on the content of GO, Gr, and HNT	Wearable biomedical devices	[97]
Triboelectric–piezoelectric	Biomechanical Ag and Cu electrodes, PTFE, Nylon, PVDF films	Self-supported structure, high electrical output performance, low-cost and large-scale fabrication process, and high stability	For low-frequency and low amplitude mechanical vibrations	Self-powered flexible pressure sensors and electronic devices	[31]
Triboelectric–piezoelectric	Mechanical vibrations Al electrodes and polyvinylidene fluoridetrifluoroethylene (PVDF-TrFE), barium titanate (BTO), and PDMS composite	Large deformations, low-cost fabrication process, high electrical output performance, and stable electrical behavior	Performance depends on the concentration of PVDF-TrFE and BTO	Self-powered sensors for body motion monitoring, functional keyboards, and self-powered electronic devices placed in vehicles, bicycles, and pavements	[99]
Triboelectric–piezoelectric	Biomechanical BTO/silicon rubber (SR) composite film	Large deformation, stretchable, and high electrical output performance	Performance depends on the BTO content	Self-powered sensors for body motion monitoring, e-skin, and flexible wearable electronics	[101]
Triboelectric–piezoelectric	Mechanical vibrations Au electrodes, antimony selenoiodide (SbSeI) nanowires, and Kapton film	Simple and cost-effective fabrication process	Low-output power density	Low-power consumption electronic devices	[111]
Triboelectric–electromagnetic	Mechanical vibrations Cu foil electrode, polypropylene (PP) film, six magnets and nine coils	Improved electrical output performance	Large volume of magnets and coils	Self-powered flexible gas and motion monitoring, and charge smartphones	[120]
Triboelectric–electromagnetic	Wind Cu electrodes, FEP film, two magnets, and four Cu coils	High electrical output performance	Large volume of electromagnetic module	Self-powered electronics devices	[129]
Triboelectric–electromagnetic	Mechanical vibrations and biomechanical Al electrodes, BTO/PVDF film, five magnets, and five coils	High electrical output performance	Performance depends on the BTO content	Self-charging power systems for outdoor search and rescue, and electronic devices in the IoT	[27]
Triboelectric–piezoelectric–pyroelectric	Mechanical vibrations, wind, and thermal PVDF nanowires-PDMS composite film and ITO electrodes	Highly transparent and flexible	Complex fabrication process	Self-powered electronics	[134]
Triboelectric–piezoelectric–electromagnetic	Mechanical vibrations and biomechanical NdFeB magnet array structure, Cu coils, beryllium bronze electrodes, lead zirconate titanate (PZT) sheets, FEP films, and Cu electrodes	Small structure, ultra-low-frequency, multi-stable, portable, and high electrical output performance	Complex assembly of the three nanogenerators modules	Self-powered portable devices for body motion monitoring, sensors for detection of bridge motions, and construction safety monitoring	[142]
Triboelectric–piezoelectric–electromagnetic	Wind PVDF/PET film, PTFE film, PVDF film, PET sheet, Al electrodes, and eight NdFeB magnets and six Cu coils	Compact and small structure, and high electrical output performance	Complex assembly of the three nanogenerators modules	Self-powered wireless environmental monitoring system in subway tunnels	[146]

## Data Availability

Request the corresponding authors of this article.

## References

[1] Keshavarz R., Shariati N. (2022). Highly sensitive and compact quad-band ambient RF energy harvester. IEEE Trans. Ind. Electron..

[2] Tao K., Chen Z., Yi H., Zhang R., Shen Q., Wu J., Tang L., Fan K., Fu Y., Miao J. (2021). Hierarchical Honeycomb-Structured Electret/Triboelectric Nanogenerator for Biomechanical and Morphing Wing Energy Harvesting. Nano-Micro Lett..

[3] Zhao T., Xu M., Xiao X., Ma Y., Li Z., Wang Z.L. (2021). Recent progress in blue energy harvesting for powering distributed sensors in ocean. Nano Energy.

[4] Tremmel S., Luo X., Rothammer B., Seynstahl A., Wang B., Rosenkranz A., Marian M., Zhu L. (2022). Evaluation of DLC, MoS2, and Ti3C2Tx thin films for triboelectric nanogenerators. Nano Energy.

[5] Niu Q., Wei H., Hsiao B.S., Zhang Y. (2022). Biodegradable silk fibroin-based bio-piezoelectric/triboelectric nanogenerators as self-powered electronic devices. Nano Energy.

[6] Lu M., Fu G., Osman N.B., Konbr U. (2021). Green energy harvesting strategies on edge-based urban computing in sustainable internet of things. Sustain. Cities Soc..

[7] Gao Y., Liu G., Bu T., Liu Y., Qi Y., Xie Y., Xu S., Deng W., Yang W., Zhang C. (2021). MXene based mechanically and electrically enhanced film for triboelectric nanogenerator. Nano Res..

[8] Islam E., Abdullah A.M., Chowdhury A.R., Tasnim F., Martinez M., Olivares C., Lozano K., Uddin M.J. (2020). Electromagnetic-triboelectric-hybrid energy tile for biomechanical green energy harvesting. Nano Energy.

[9] Shaukat R.A., Saqib Q.M., Khan M.U., Chougale M.Y., Bae J. (2021). Bio-waste sunflower husks powder based recycled triboelectric nanogenerator for energy harvesting. Energy Reports.

[10] Niu Z., Cheng W., Cao M., Wang D., Wang Q., Han J., Long Y., Han G. (2021). Recent advances in cellulose-based flexible triboelectric nanogeneratorss. Nano Energy.

[11] Nguyen Q.-T., Ahn K.-K.K. (2021). Fluid-Based Triboelectric Nanogenerators: A Review of Current Status and Applications. Int. J. Precis. Eng. Manuf. Green Technol..

[12] Sanad M.F., Shalan A.E., Abdellatif S.O., Serea E.S.A., Adly M.S., Ahsan M. (2020). Thermoelectric Energy Harvesters: A Review of Recent Developments in Materials and Devices for Different Potential Applications. Top. Curr. Chem. (Z).

[13] Karan S.K., Maiti S., Lee J.H., Mishra Y.K., Khatua B.B., Kim J.K. (2020). Recent Advances in Self-Powered Tribo-/Piezoelectric Energy Harvesters: All-In-One Package for Future Smart Technologies. Adv. Funct. Mater..

[14] Nie W. (2022). A sliding hybrid triboelectric-electromagnetic nanogenerator with staggered electrodes for human motion posture. Energy Rep..

[15] Sahu M., Hajra S., Panda S., Rajaitha M., Panigrahi B.K., Rubahn H.-G., Mishra Y.K., Kim H.J. (2022). Waste textiles as the versatile triboelectric energy-harvesting platform for self-powered applications in sports and athletics. Nano Energy.

[16] Pang Y., Zhu X., Lee C., Liu S. (2022). Triboelectric nanogenerator as next-generation self-powered sensor for cooperative vehicle-infrastructure system. Nano Energy.

[17] Bhatia D., Lee K.-S., Niazi M.U.K., Park H.-S. (2022). Triboelectric nanogenerator integrated origami gravity support device for shoulder rehabilitation using exercise gaming. Nano Energy.

[18] Fang L., Zheng Q., Hou W., Zheng L., Li H. (2022). A self-powered vibration sensor based on the coupling of triboelectric nanogenerator and electromagnetic generator. Nano Energy.

[19] Shi S., Jiang Y., Xu Q., Zhang J., Zhang Y., Li J., Xie Y., Cao Z.-P. (2022). A self-powered triboelectric multi-information motion monitoring sensor and its application in wireless real-time control. Nano Energy.

[20] Miskovsky N.M., Cutler P.H., Mayer A., Weiss B.L., Willis B., Sullivan T.E., Lerner P.B. (2012). Nanoscale devices for rectification of high frequency Radiation from the infrared through the visible: A new approach. J. Nanotech..

[21] Donchev E., Pang J.S., Gammon P.M., Centeno A., Xie F., Petrov P.K., Breeze J.D., Ryan M.P., Riley D.J., Alford N. (2014). The rectenna device: From theory to practice (a review). MRS Energy Sustain..

[22] Mupparapu R., Cunha J., Tantussi F., Jacassi A., Summerer L., Patrini M., Giugni A., Maserati L., Alabastri A., Garoli D. (2022). High-Frequency Light Rectification by Nanoscale Plasmonic Conical Antenna in Point-Contact-Insulator-Metal Architecture. Adv. Energy Mater..

[23] Shanawani M., Masotti D., Costanzo A. (2017). THz Rectennas and Their Design Rules. Electronics.

[24] Byrnes S.J., Blanchard R., Capasso F. (2014). Harvesting renewable energy from Earth’s mid-infrared emissions. Proc. Natl. Acad. Sci. USA.

[25] Joshi S., Moddel G. (2015). Rectennas at optical frequencies: How to analyze the response. J. Applied Phys..

[26] Zhao J., Cong Z., Hu J., Lu H., Wang L., Wang H., Malyi O.I., Pu X., Zhang Y., Shao H. (2022). Regulating zinc electroplating chemistry to achieve high energy coaxial fiber Zn ion supercapacitor for self-powered textile-based monitoring system. Nano Energy.

[27] Zhang Y., Gao X., Zhang Y., Gui J., Sun C., Zheng H., Guo S. (2022). High-efficiency self-charging power systems based on performance-enhanced hybrid nanogenerators and asymmetric supercapacitors for outdoor search and rescue. Nano Energy.

[28] Yu Z., Zhang Y., Wang Y., Zheng J., Fu Y., Chen D., Wang G., Cui J., Yu S., Zheng L. (2022). Integrated piezo-tribo hybrid acoustic-driven nanogenerator based on porous MWCNTs/PVDF-TrFE aerogel bulk with embedded PDMS tympanum structure for broadband sound energy harvesting. Nano Energy.

[29] Li R., Wei X., Shi Y., Yuan Z., Wang B., Xu J., Wang L., Wu Z., Wang Z.L. (2022). Low-grade heat energy harvesting system based on the shape memory effect and hybrid triboelectric-electromagnetic nanogenerator. Nano Energy.

[30] Banerjee S., Bairagi S., Ali S.W. (2022). A lead-free flexible piezoelectric-triboelectric hybrid nanogenerator composed of uniquely designed PVDF/KNN-ZS nanofibrous web. Energy.

[31] Song C., Xia K., Xu Z. (2022). A self-supported structure hybrid triboelectric/piezoelectric nanogenerator for biomechanical energy harvesting and pressure sensing. Microelectron. Eng..

[32] Zhao Z., Dai Y., Dou S.X., Liang J. (2021). Flexible nanogenerators for wearable electronic applications based on piezoelectric materials. Mater. Today Energy.

[33] Hinchet R., Seung W., Kim S.W. (2015). Recent Progress on Flexible Triboelectric Nanogenerators for SelfPowered Electronics. ChemSusChem.

[34] Quelen A., Morel A., Gasnier P., Grézaud R., Monfray S., Pillonnet G. A 30nA quiescent 80nW-to-14mW power-range shock-optimized SECE-based piezoelectric harvesting interface with 420% harvested-energy improvement. Proceedings of the 2018 IEEE International Solid-State Circuits Conference—(ISSCC).

[35] Hehn T., Hagedorn F., Maurath D., Marinkovic D., Kuehne I., Frey A., Manoli Y. (2012). A Fully Autonomous Integrated Interface Circuit for Piezoelectric Harvesters. IEEE J. Solid-State Circuits.

[36] Mahapatra A., Ajimsha R.S., Misra P. (2022). Oxygen annealing induced enhancement in output characteristics of ZnO based flexible piezoelectric nanogenerators. J. Alloys Compd..

[37] Huang X., Wang Y., Zhang X. (2022). Ultrarobust, hierarchically anisotropic structured piezoelectric nanogenerators for self-powered sensing. Nano Energy.

[38] Veeralingam S., Bharti D.K., Badhulika S. (2022). Lead-free PDMS/PPy based low-cost wearable piezoelectric nanogenerator for self-powered pulse pressure sensor application. Mater. Res. Bull..

[39] Sahu M., Hajra S., Jadhav S., Panigrahi B.K., Dubal D., Kim H.J. (2022). Bio-waste composites for cost-effective self-powered breathing patterns monitoring: An insight into energy harvesting and storage properties. Sustain. Mater. Technol..

[40] Sarkar L., Sushma M.V., Yalagala B.P., Rengan A.K., Singh S.G., Vanjari S.R.K. (2022). ZnO nanoparticles embedded silk fibroin-a piezoelectric composite for nanogenerator applications. Nanotechnology.

[41] Pusty M., Shirage P.M. (2022). Insights and perspectives on graphene-PVDF based nanocomposite materials for harvesting mechanical energy. J. Alloys Compd..

[42] Tan P., Xi Y., Chao S., Jiang D., Liu Z., Fan Y., Li Z. (2022). An Artificial Intelligence-Enhanced Blood Pressure Monitor Wristband Based on Piezoelectric Nanogenerator. Biosensors.

[43] Mahmud M.A.P., Adhikary P., Zolfagharian A., Adams S., Kaynak A., Kouzani A.Z. (2022). Advanced design, fabrication, and applications of 3D-printable piezoelectric nanogenerators. Electron. Mater. Lett..

[44] Shi S., Pan Z., Cheng Y., Zhai Y., Zhang Y., Ding X., Liu J., Zhai J., Xu J. (2022). Three-dimensional polypyrrole induced high-performance flexible piezoelectric nanogenerators for mechanical energy harvesting. Comp. Sci. Technol..

[45] Pan Q., Wang B., Zhang L., Li Z., Yang Z. (2022). Whisk-inspired Motion Converter for Ocean Wave Energy Harvesting. IEEE/ASME Trans. Mechatron..

[46] Wang J., Pan L., Guo H., Zhang B., Zhang R., Wu Z., Wu C., Yang L., Liao R., Wang Z.L. (2019). Rational Structure Optimized Hybrid Nanogenerator for Highly Efficient Water Wave Energy Harvesting. Adv. Energy Mater..

[47] Wu Y., Zeng Q., Tang Q., Liu W., Liu G., Zhang Y., Wu J., Hu C., Wang X. (2020). A teeterboard-like hybrid nanogenerator for efficient harvesting of low-frequency ocean wave energy. Nano Energy.

[48] Zhang X., Yang Q., Ji P., Wu Z., Li Q., Yang H., Li X., Zheng G., Xi Y., Wang Z.L. (2022). Modeling of liquid-solid hydrodynamic water wave energy harvesting system based on triboelectric nanogenerator. Nano Energy.

[49] Zaw N.Y.W., Yun J., Goh T.S., Kim I., Kim Y., Lee J.S., Kim D. (2022). All-polymer waterproof triboelectric nanogenerator towards blue energy harvesting and self-powered human motion detection. Energy.

[50] Zhou H., Dong J., Liu H., Zhu L., Xu C., He X., Zhang S., Song Q. (2022). The coordination of displacement and conduction currents to boost the instantaneous power output of a water-tube triboelectric nanogenerator. Nano Energy.

[51] Li W., Wan L., Lin Y., Liu G., Qu H., Wen H., Ding J., Ning H., Yao H. (2022). Synchronous nanogenerator with intermittent sliding friction self-excitation for water wave energy harvesting. Nano Energy.

[52] Zhang Z., Hu Z., Wang Y., Wang Y., Zhang Q., Liu D., Wang H., Xu M. (2022). Multi-Tunnel Triboelectric Nanogenerator for Scavenging Mechanical Energy in Marine Floating Bodies. J. Mar. Sci. Eng..

[53] Wang X., Shi Y., Yang P., Tao X., Li S., Lei R., Liu Z., Wang Z.L., Chen X. (2022). Fish-Wearable Data Snooping Platform for Underwater Energy Harvesting and Fish Behavior Monitoring. Small.

[54] Xu L., Jiang T., Lin P., Shao J.J., He C., Zhong W., Chen X.Y., Wang Z.L. (2018). Coupled Triboelectric Nanogenerator Networks for Efficient Water Wave Energy Harvesting. ACS Nano.

[55] Wen H., Yang P., Liu G., Xu S., Yao H., Li W., Qu H., Ding J., Li J., Wan L. (2022). Flower-like triboelectric nanogenerator for blue energy harvesting with six degrees of freedom. Nano Energy.

[56] Wang A., Chen J., Wang L., Han J., Su W., Li A., Liu P., Duan L., Xu C., Zeng Z. (2022). Numerical analysis and experimental study of an ocean wave tetrahedral triboelectric nanogenerator. Appl. Energy.

[57] Qu Z., Huang M., Chen C., An Y., Liu H., Zhang Q., Wang X., Liu Y., Yin W., Li X. (2022). Spherical triboelectric nanogenerator based on eccentric structure for omnidirectional low frequency water wave energy harvesting. Adv. Funct. Mater..

[58] Xu S., Liu G., Wang J., Wen H., Cao S., Yao H., Wan L., Wang Z.L. (2021). Interaction between water wave and geometrical structures of floating triboelectric nanogenerators. Adv. Energy Mater..

[59] Feng Y., Han J., Xu M., Liang X., Jiang T., Li H., Wang Z.L. (2021). Blue energy for green hydrogen fuel: A self-powered electrochemical conversion driven by triboelectric nanogenerators. Adv. Energy Mater..

[60] Zheng F., Sun Y., Wei X., Chen J., Yuan Z., Jin X., Tao L., Wu Z. (2021). A hybridized water wave energy harvester with a swing magnetic structure toward intelligent fishing ground. Nano Energy.

[61] Torres F.G., Troncoso O.P., De-la-Torre G.E. (2022). Hydrogel-based triboelectric nanogenerators: Properties, performance, and applications. Int. J. Energy Res..

[62] Chen B., Wang Z.L. (2022). Toward a new era of sustainable energy: Advanced triboelectric nanogenerator for harvesting high entropy energy. Small.

[63] Bukhari M.U., Khan A., Maqbool K.Q., Arshad A., Riaz K., Bermak A. (2022). Waste to energy: Facile, low-cost and environment-friendly triboelectric nanogenerators using recycled plastic and electronic wastes for self-powered portable electronics. Energy Rep..

[64] Zhang J., Hu Y., Lin X., Qian X., Zhang L., Zhou J., Lu A. (2022). High-performance triboelectric nanogenerator based on chitin for mechanical-energy harvesting and self-powered sensing. Carbohydr. Polym..

[65] Gravesen J., Willatzen M., Shao J., Wang Z.L. (2022). Energy optimization of a mirror-symmetric spherical triboelectric nanogenerator. Adv. Funct. Mater..

[66] Wu H., Wang J., Wu Z., Kang S., Wei X., Wang H., Luo H., Yang L., Liao R., Wang Z.L. (2022). Multi-parameter optimized triboelectric nanogenerator based self-powered sensor network for broadband aeolian vibration online-monitoring of transmission lines. Adv. Energy Mater..

[67] Wang J., Jiang Z., Sun W., Xu X., Han Q., Chu F. (2022). Yoyo-ball inspired triboelectric nanogenerators for harvesting biomechanical energy. Appl. Energy.

[68] Mathew A.A., Vivekanandan S. (2022). Design and Simulation of Single-Electrode Mode Triboelectric Nanogenerator-Based Pulse Sensor for Healthcare Applications Using COMSOL Multiphysics. Energy Technol..

[69] Zheng Z., Xia J., Wang B., Guo Y. (2022). Hierarchically designed nanocomposites for triboelectric nanogenerator toward biomechanical energy harvester and smart home system. Nano Energy.

[70] Sun Z., Yang W., Chen P., Zhang Y., Wang X., Hu Y. (2022). Effects of PDMS Base/Agent Ratios and Texture Sizes on the Electrical Performance of Triboelectric Nanogenerators. Adv. Mater. Interfaces.

[71] Jiang C., Lai C.L., Xu B., So M.Y., Li Z. (2022). Fabric-rebound triboelectric nanogenerators with loops and layered structures for energy harvesting and intelligent wireless monitoring of human motions. Nano Energy.

[72] Wang Z.L. (2014). Triboelectric nanogenerators as new energy technology and self-powered sensors-principles, problems and perspectives. Faraday Discuss..

[73] Wang Z. (2007). Nanopiezotronics. Adv. Mater..

[74] Wang Z.L. (2008). Towards Self-Powered Nanosystems: From Nanogenerators to Nanopiezotronics. Adv. Funct. Mater..

[75] Wang Z.L. (2007). The new field of nanopiezotronics. Mater. Today.

[76] Yang Y., Yang Y. (2020). Pyroelectric and Thermoelectric Nanogenerators. Hybridized and Coupled Nanogenerators.

[77] Klochko N.P., Klepikova K.S., Kopach V.R., Tyukhov I.I., Starikov V.V., Sofronov D.S., Khrypunova I.V., Zhadan D.O., Petrushenko S.I., Dukarov S.V. (2019). Development of semi-transparent ZnO/FTO solar thermoelectric nanogenerator for energy efficient glazing. Sol. Energy.

[78] Khoso N.A., Xu G., Xie J., Sun T., Wang J. (2021). The fabrication of a graphene and conductive polymer nanocomposite-coated highly flexible and washable woven thermoelectric nanogenerator. Mater. Adv..

[79] Khoso N.A., Ahmed A., Deb H., Tian S., Jiao X., Gong X.Y., Wang J. (2019). Controlled template-free in-situ polymerization of PEDOT for enhanced thermoelectric performance on textile substrate. Org. Electron..

[80] Feng R., Tang F., Zhang N., Wang X. (2019). Flexible, High-Power Density, Wearable Thermoelectric Nanogenerator and Self-Powered Temperature Sensor. ACS Appl. Mater. Interfaces.

[81] He M., Lin Y.-J., Chiu C.M., Yang W., Zhang B., Yun D., Xie Y., Lin Z.H. (2018). A flexible photo-thermoelectric nanogenerator based on MoS2/PU photothermal layer for infrared light harvesting. Nano Energy.

[82] Fan Z., Zhang Y., Pan L., Ouyang J., Zhang Q. (2021). Recent developments in flexible thermoelectrics: From materials to devices. Renew. Sust. Energ. Rev..

[83] Xie Y., Chou T.M., Yang W., He M., Zhao Y., Li N., Lin Z.-H. (2017). Flexible thermoelectric nanogenerator based on the MoS_2_/graphene nanocomposite and its application for a self-powered temperature sensor. Semicond. Sci. Technol..

[84] Huang X.-L., Ao D.-W., Chen T.-B., Chen Y.-X., Li F., Chen S., Liang G.-X., Zhang X.-H., Zheng Z.-H., Fan P. (2021). High performance copper selenide thermoelectric thin films for flexible thermoelectric application. Mater. Today Energy.

[85] Xu C., Yang S., Li P., Wang H., Li H., Liu Z. (2022). Wet-spun PEDOT:PSS/CNT composite fibers for wearable thermoelectric energy harvesting. Compos. Commun..

[86] Palaporn D., Mongkolthanaruk W., Tanusilp S.-A., Kurosaki K., Pinitsoontorn S. (2022). A simple method for fabricating flexible thermoelectric nanocomposites based on bacterial cellulose nanofiber and Ag_2_Se. Appl. Phys. Lett..

[87] Zhang X., Shiu B.-C., Li T.T., Liu X., Ren H.-T., Wang Y., Lou C.-W., Lin J.-H. (2021). Synergistic work of photo-thermoelectric and hydroelectric effects of hierarchical structure photo-thermoelectric textile for solar energy harvesting and solar steam generation simultaneously. Chem. Eng. J..

[88] Wang Y., Zhu W., Deng Y., Zhu P., Yu Y., Hu S., Zhang R. (2022). High-sensitivity self-powered temperature/pressure sensor based on flexible Bi-Te thermoelectric film and porous microconed elastomer. J. Mater. Sci. Technol..

[89] Yuan J.F., Zhu R., Li G.Z. (2020). Self-Powered Electronic Skin with Multisensory Functions Based on Thermoelectric Conversion. Adv. Mater. Technol..

[90] Wang Y., Zhu W., Deng Y., Fu B., Zhu P., Yu Y., Li J., Guo J. (2020). Self-powered wearable pressure sensing system for continuous healthcare monitoring enabled by flexible thin-film thermoelectric generator. Nano Energy.

[91] Gokana M.R., Wu C.-M., Motora K.G., Qi J.Y., Yen W.-T. (2022). Effects of patterned electrode on near infrared light-triggered cesium tungsten bronze/poly(vinylidene)fluoride nanocomposite-based pyroelectric nanogenerator for energy harvesting. J. Power Sources.

[92] Mistewicz K. (2022). Pyroelectric Nanogenerator Based on an SbSI–TiO_2_ Nanocomposite. Sensors.

[93] Feng Y., Zhang Y., Wang Y., Wang Z. (2018). Frequency response characteristics of pyroelectric effect in p-n junction UV detectors. Nano Energy.

[94] Korkmaz S., Kariper İ.A. (2021). Pyroelectric nanogenerators (PyNGs) in converting thermal energy into electrical energy: Fundamentals and current status. Nano Energy.

[95] Ali I., Hassan G., Shuja A. (2022). Fabrication of self-healing hybrid nanogenerators based on polyurethane and ZnO for harvesting wind energy. J Mater. Sci. Mater. Electron..

[96] Mahanty B., Ghosh S.K., Maity K., Roy K., Sarkar S., Mandal D. (2021). All-fiber pyro- and piezo-electric nanogenerator for IoT based self-powered health-care monitoring. Mater. Adv..

[97] Abbasipour M., Khajavi R., Yousefi A.A., Yazdanshenas M.E., Razaghian F., Akbarzadeh A. (2019). Improving piezoelectric and pyroelectric properties of electrospun PVDF nanofibers using nanofillers for energy harvesting application. Polym. Adv. Technol..

[98] Zhu L., Lin P., Chen B., Wang L., Chen L., Li D., Wang Z.L. (2018). Piezo-phototronic and pyro-phototronic effects to enhance Cu(In, Ga)Se2 thin film solar cells. Nano Res..

[99] Liu X., Liu Y., Cheng T., Gao Y., Yang Z. (2022). A high performing piezoelectric and triboelectric nanogenerator based on a large deformation of the novel lantern-shaped structure. Nano Energy.

[100] Wang Z., Liu Z., Zhao G., Zhang Z., Zhao X., Wan X., Zhang Y., Wang Z.L., Li L. (2022). Stretchable Unsymmetrical Piezoelectric BaTiO3 Composite Hydrogel for Triboelectric Nanogenerators and Multimodal Sensors. ACS Nano.

[101] Hou X., Zhong J., Yang C., Yang Y., He J., Mu J., Geng W., Chou X. (2022). A high-performance, single-electrode and stretchable piezo-triboelectric hybrid patch for omnidirectional biomechanical energy harvesting and motion monitoring. J. Mater..

[102] Joo H., Lee K.Y., Lee J. (2022). Piezo/Triboelectric Effect Driven Self-Powered Gas Sensor for Environmental Sensor Networks. Energy Technol..

[103] Negedu S.D., Tromer R., Gowda C.C., Woellner C.F., Olu F.E., Roy A.K., Pandey P., Galvao D.S., Ajayan P.M., Kumbhakar P. (2022). Two-dimensional cobalt telluride as a piezo-tribogenerator. Nanoscale.

[104] Zhang J.-H., Zhou Z., Li J., Shen B., Zhu T., Gao X., Tao R., Guo X., Hu X., Shi Y. (2022). Coupling Enhanced Performance of Triboelectric–Piezoelectric Hybrid Nanogenerator Based on Nanoporous Film of Poly(vinylidene fluoride)/BaTiO3 Composite Electrospun Fibers. ACS Mater. Lett..

[105] Nazar A.M., Egbe K.I., Jiao P. (2022). Hybrid Piezoelectric and Triboelectric Nanogenerators for Energy Harvesting and Walking Sensing. Energy Technol..

[106] Du M., Cao Y., Qu X., Xue J., Zhang W., Pu X., Shi B., Li Z. (2022). Hybrid Nanogenerator for Biomechanical Energy Harvesting, Motion State Detection, and Pulse Sensing. Adv. Mater. Technol..

[107] Kao F.-C., Ho H.-H., Chiu P.-Y., Hsieh M.-K., Liao J.-C., Lai P.-L., Huang Y.-F., Dong M.-Y., Tsai T.T., Lin Z.-H. (2022). Self-assisted wound healing using piezoelectric and triboelectric nanogenerators. Sci. Technol. Adv. Mater..

[108] Manchi P., Graham S.A., Patnam H., Paranjape M.V., Yu J.S. (2022). rGO-ZnSnO_3_ Nanostructure-Embedded Triboelectric Polymer-Based Hybridized Nanogenerators. Adv. Mater. Technol..

[109] Zhu Y., Sun F., Jia C., Zhao T., Mao Y. (2022). A Stretchable and Self-Healing Hybrid Nano-Generator for Human Motion Monitoring. Nanomaterials.

[110] García-Casas X., Ghaffarinejad A., Aparicio F.J., Castillo-Seoane J., López-Santos C., Espinós J.P., Cotrino J., Sánchez-Valencia J.R., Barranco A., Borrás A. (2022). Plasma engineering of microstructured piezo—Triboelectric hybrid nanogenerators for wide bandwidth vibration energy harvesting. Nano Energy.

[111] Toroń B., Mistewicz K., Jesionek M., Kozioł M., Zubko M., Stróż D. (2022). A new hybrid piezo/triboelectric SbSeI nanogenerator. Energy.

[112] Li X., Ji D., Yu B., Ghosh R., He J., Qin X., Ramakrishna S. (2021). Boosting piezoelectric and triboelectric effects of PVDF nanofiber through carbon-coated piezoelectric nanoparticles for highly sensitive wearable sensors. Chem. Eng. J..

[113] Yang Z., Zhu Z., Chen Z., Liu M., Zhao B., Liu Y., Cheng Z., Wang S., Yang W., Yu T. (2021). Recent Advances in Self-Powered Piezoelectric and Triboelectric Sensors: From Material and Structure Design to Frontier Applications of Artificial Intelligence. Sensors.

[114] Yang X., Li P., Wu B., Li H., Zhou G. (2021). A flexible piezoelectric-triboelectric hybrid nanogenerator in one structure with dual doping enhancement effects. Curr. Appl. Phys..

[115] Lee T., Kim I., Kim D. (2021). Flexible Hybrid Nanogenerator for Self-Powered Weather and Healthcare Monitoring Sensor. Adv. Electron. Mater..

[116] Singh H.H., Khare N. (2021). A ferroelectric nanocomposite-film-based device for harvesting energy from water droplets using both piezoelectric and triboelectric effects. Nanotechnology.

[117] Hajra S., Padhan A.M., Sahu M., Alagarsamy P., Lee K., Kim H.J. (2021). Lead-free flexible Bismuth Titanate-PDMS composites: A multifunctional colossal dielectric material for hybrid piezo-triboelectric nanogenerator to sustainably power portable electronics. Nano Energy.

[118] Gai Y., Bai Y., Cao Y., Wang E., Xue J., Qu X., Liu Z., Luo D., Li Z. (2022). A Gyroscope Nanogenerator with Frequency Up-Conversion Effect for Fitness and Energy Harvesting. Small.

[119] Mu J., He H., Song J., He J., Hou X., Han X., Feng C., Zou J., Yu J., Chou X. (2022). Functional structure enhanced synergistic sensing from triboelectric–electromagnetic hybrid nanogenerator for self-powered rotating speed monitoring. Energy Rep..

[120] Wang D., Zhang D., Tang M., Zhang H., Chen F., Wang T., Li Z., Zhao P. (2022). Rotating triboelectric-electromagnetic nanogenerator driven by tires for self-powered MXene-based flexible wearable electronics. Chem. Eng. J..

[121] Yu D., Sun C., Wang K., Yin S., Sun L., Chen H., Kong F. (2022). A novel direct-driven triboelectric–electromagnetic hybridized wave energy converter for buoy power supply. Appl. Nanosci..

[122] Cho H., Kim I., Park J., Kim D. (2022). A waterwheel hybrid generator with disk triboelectric nanogenerator and electromagnetic generator as a power source for an electrocoagulation system. Nano Energy.

[123] He J., Fan X., Zhao D., Cui M., Han B., Hou X., Chou X. (2022). A high-efficient triboelectric-electromagnetic hybrid nanogenerator for vibration energy harvesting and wireless monitoring. Sci. China Inf. Sci..

[124] Hu Y., Wang X., Qin Y., Li Z., Wang C., Wu H. (2022). A robust hybrid generator for harvesting vehicle suspension vibration energy from random road excitation. Appl. Energy.

[125] Chen Y., Jie Y., Zhu J., Lu Q., Cheng Y., Cao X., Wang Z.L. (2022). Hybridized triboelectric-electromagnetic nanogenerators and solar cell for energy harvesting and wireless power transmission. Nano Res..

[126] Mu J., Zou J., Song J., He J., Hou X., Yu J., Han X., Feng C., He H., Chou X. (2022). Hybrid enhancement effect of structural and material properties of the triboelectric generator on its performance in integrated energy harvester. Energy Convers. Manag..

[127] Kong F., Yin S., Sun C., Yang C., Chen H., Liu H. (2022). Design and optimization of a maglev electromagnetic–triboelectric hybrid energy converter for supplying power to intelligent sensing equipment. Sustain. Energy Fuels.

[128] Hong H., Yang X., Cui H., Zheng D., Wen H., Huang R., Liu L., Duan J., Tang Q. (2022). Self-powered seesaw structured spherical buoys based on a hybrid triboelectric–electromagnetic nanogenerator for sea surface wireless positioning. Energy Environ. Sci..

[129] Li X., Gao Q., Cao Y., Yang Y., Liu S., Wang Z.L. (2022). Tinghai Cheng. Optimization strategy of wind energy harvesting via triboelectric-electromagnetic flexible cooperation. Appl. Energy.

[130] Xin C., Guo H., Shen F., Peng Y., Xie S., Li Z., Zhang Q. (2022). A Hybrid Generator with Electromagnetic Transduction for Improving the Power Density of Triboelectric Nanogenerators and Scavenging Wind Energy. Adv. Mater. Technol..

[131] Zhao T., Niu B., Xie G., Hu C., Liu B., Xu M., Ma Y. (2022). A High Output Triboelectric–Electromagnetic Hybrid Generator Based on In-Phase Parallel Connection. Adv. Mater. Technol..

[132] Zhang B., Zhang S., Li W., Gao Q., Zhao D., Wang Z.L., Cheng T. (2021). Self-Powered Sensing for Smart Agriculture by Electromagnetic–Triboelectric Hybrid Generator. ACS Nano.

[133] Askari H., Xu N., Barbosa B.H.G., Huang Y., Chen L., Khajepour A., Chen H., Wang Z.L. (2022). Intelligent systems using triboelectric, piezoelectric, and pyroelectric nanogenerators. Mater. Today.

[134] Wang S., Wang Z.L., Yang Y. (2016). A One-Structure-Based Hybridized Nanogenerator for Scavenging Mechanical and Thermal Energies by Triboelectric–Piezoelectric–Pyroelectric Effects. Adv. Mater..

[135] Zhang H., Zhang S., Yao G., Huang Z., Xie Y., Su Y., Yang W., Zheng C., Lin Y. (2015). Simultaneously Harvesting Thermal and Mechanical Energies based on Flexible Hybrid Nanogenerator for Self-Powered Cathodic Protection. ACS Appl. Mater. Interfaces.

[136] Zi Y., Lin L., Wang J., Wang S., Chen J., Fan X., Yang P.-K., Yi F., Wang Z.L. (2015). Triboelectric–Pyroelectric–Piezoelectric Hybrid Cell for High-Efficiency Energy-Harvesting and Self-Powered Sensing. Adv. Mater..

[137] Khan A.A., Saritas R., Rana M.M., Tanguy N., Zhu W., Mei N., Kokilathasan S., Rassel S., Leonenko Z., Yan N. (2022). Performance-Improved Highly Integrated Uniaxial Tristate Hybrid Nanogenerator for Sustainable Mechanical Energy Harvesting. ACS Appl. Mater. Interfaces.

[138] Tang G., Wang Z., Hu X., Wu S., Xu B., Li Z., Yan X., Xu F., Yuan D., Li P. (2022). A Non-Resonant Piezoelectric–Electromagnetic–Triboelectric Hybrid Energy Harvester for Low-Frequency Human Motions. Nanomaterials.

[139] Zhao L.-C., Zou H.-X., Zhao Y.-J., Wu Z.-Y., Liu F.-R., Wei K.-X., Zhang W.-M. (2022). Hybrid energy harvesting for self-powered rotor condition monitoring using maximal utilization strategy in structural space and operation process. Appl. Energy.

[140] Zhang C., Yuan W., Zhang B., Yang O., Liu Y., He L., Wang J., Wang Z.L. (2022). High Space Efficiency Hybrid Nanogenerators for Effective Water Wave Energy Harvesting. Adv. Funct. Mater..

[141] Xue F., Chen L., Li C., Ren J., Yu J., Hou X., Geng W., Mu J., He J., Chou X. (2022). A static-dynamic energy harvester for a self-powered ocean environment monitoring application. Sci. China Technol. Sci..

[142] Wang C., Lai S.-K., Wang J.-M., Feng J.-J., Ni Y.-Q. (2021). An ultra-low-frequency, broadband and multi-stable tri-hybrid energy harvester for enabling the next-generation sustainable power. Appl. Energy.

[143] Xue X., Zhang Z., Wu B., He S., Wang Q., Zhang W., Bi R., Cui J., Zheng Y., Xue C. (2021). Coil-levitated hybrid generator for mechanical energy harvesting and wireless temperature and vibration monitoring. Sci. China Technol. Sci..

[144] Ma T., Gao Q., Li Y., Wang Z., Lu X., Cheng T. (2020). An Integrated Triboelectric–Electromagnetic–Piezoelectric Hybrid Energy Harvester Induced by a Multifunction Magnet for Rotational Motion. Adv. Eng. Mater..

[145] Rodrigues C., Gomes A., Ghosh A., Pereira A., Ventura J. (2019). Power-generating footwear based on a triboelectric-electromagnetic-piezoelectric hybrid nanogenerator. Nano Energy.

[146] Rahman M.T., Salauddin M., Maharjan P., Rasel M.S., Cho H., Park J.Y. (2019). Natural wind-driven ultra-compact and highly efficient hybridized nanogenerator for self-sustained wireless environmental monitoring system. Nano Energy.

[147] Du X., Zhao S., Xing Y., Li N., Wang J., Zhang X., Cao R., Liu Y., Yuan Z., Yin Y. (2018). Hybridized Nanogenerators for Harvesting Vibrational Energy by Triboelectric–Piezoelectric–Electromagnetic Effects. Adv. Mater. Technol..

[148] He J., Wen T., Qian S., Zhang Z., Tian Z., Zhu J., Mu J., Hou X., Geng W., Cho J. (2018). Triboelectric-piezoelectric-electromagnetic hybrid nanogenerator for high-efficient vibration energy harvesting and self-powered wireless monitoring system. Nano Energy.

[149] Liu Y., Sun N., Liu J., Wen Z., Sun X., Lee S.-T., Sun B. (2018). Integrating a Silicon Solar Cell with a Triboelectric Nanogenerator via a Mutual Electrode for Harvesting Energy from Sunlight and Raindrops. ACS Nano.

[150] Im B., Lee S.-K., Kang G., Moon J., Byun D., Cho D.-H. (2022). Electrohydrodynamic jet printed silver-grid electrode for transparent raindrop energy-based triboelectric nanogenerator. Nano Energy.

[151] Zheng Y., Liu T., Wu J., Xu T., Wang X., Han X., Cui H., Xu X., Pan C., Li X. (2022). Energy Conversion Analysis of Multilayered Triboelectric Nanogenerators for Synergistic Rain and Solar Energy Harvesting. Adv. Mater..

[152] Wu Y., Qu J., Chu P.K., Shin D.-M., Luo Y., Feng S.-P. (2021). Hybrid photovoltaic-triboelectric nanogenerators for simultaneously harvesting solar and mechanical energies. Nano Energy.

[153] Sivasubramanian R., Vaithilingam C.A., Indira S.S., Paiman S., Misron N., Abubakar S. (2021). A review on photovoltaic and nanogenerator hybrid system. Mater. Today Energy.

[154] Yang D., Ni Y., Su H., Shi Y., Liu Q., Chen X., He D. (2021). Hybrid energy system based on solar cell and self-healing/self-cleaning triboelectric nanogenerator. Nano Energy.

[155] Hu S., Han J., Shi Z., Chen K., Xu N., Wang Y., Zheng R., Tao Y., Sun Q., Wang Z.L. (2022). Biodegradable, Super-Strong, and Conductive Cellulose Macrofibers for Fabric-Based Triboelectric Nanogenerator. Nano-Micro Lett..

[156] Zhao J., Mu J., Cui H., He W., Zhang L., He J., Gao X., Li Z., Hou X., Chou X. (2021). Hybridized Triboelectric-Electromagnetic Nanogenerator for Wind Energy Harvesting to Realize Real-Time Power Supply of Sensor Nodes. Adv. Mater. Technol..

[157] Wu Y., Zhong X., Wang X., Yang Y., Wang Z.L. (2014). Hybrid energy cell for simultaneously harvesting wind, solar, and chemical energies. Nano Res..

[158] Denning D., Kilpatrick J.I., Fukada E., Zhang N., Habelitz S., Fertala A., Gilchrist M.D., Zhang Y., Tofail S.A.M., Rodriguez B.J. (2017). Piezoelectric Tensor of Collagen Fibrils Determined at the Nanoscale. ACS Biomater. Sci. Eng..

[159] Knoblich M., Anderson B., Latshaw D. (2005). Analyses of tomato peel and seed byproducts and their use as a source of carotenoids. J. Sci. Food Agric..

[160] Saqib Q.M., Khan M.U., Song H., Chougale M.Y., Shaukat R.A., Kim J., Bae J., Choi M.J., Kim S.C., Kwon O. (2021). Natural Hierarchically Structured Highly Porous Tomato Peel Based Tribo- and Piezo-Electric Nanogenerator for Efficient Energy Harvesting. Adv. Sustain. Syst..

[161] Mao Y., Zhao P., McConohy G., Yang H., Tong Y., Wang X. (2014). Sponge-Like Piezoelectric Polymer Films for Scalable and Integratable Nanogenerators and Self-Powered Electronic Systems. Adv. Energy Mater..

[162] Zhu Y., Xia Y., Wu M., Guo W., Jia C., Wang X. (2022). Wearable, freezing-tolerant, and self-powered electroluminescence system for long-term cold-resistant displays. Nano Energy.

[163] Karan S.K., Maiti S., Paria S., Maitra A., Si S.K., Kim J.K., Khatua B.B. (2018). A new insight towards eggshell membrane as high energy conversion efficient bio-piezoelectric energy harvester. Mater. Today Energy.

[164] Zhang M., Du H., Liu K., Nie S., Xu T., Zhang X., Si C. (2021). Fabrication and applications of cellulose-based nanogenerators. Adv. Compos. Hybrid Mater..

[165] Ghosh S.K., Mandal D. (2016). Efficient natural piezoelectric nanogenerator: Electricity generation from fish swim bladder. Nano Energy.

[166] Karan S.K., Maiti S., Kwon O., Paria S., Maitra A., Si S.K., Kim Y., Kim J.K., Khatua B.B. (2018). Nature driven spider silk as high energy conversion efficient bio-piezoelectric nanogenerator. Nano Energy.

[167] Saqib Q.M., Shaukat R.A., Khan M.U., Chougale M., Bae J. (2020). Biowaste Peanut Shell Powder-Based Triboelectric Nanogenerator for Biomechanical Energy Scavenging and Sustainably Powering Electronic Supplies. ACS Appl. Electron. Mater..

[168] Chougale M.Y., Saqib Q.M., Khan M.U., Shaukat R.A., Kim J., Bae J. (2021). Novel Recycled Triboelectric Nanogenerator Based on Polymer-Coated Trash Soda Can for Clean Energy Harvesting. Adv. Sustain. Syst..

[169] Jiang W., Li H., Liu Z., Li Z., Tian J.J., Shi B.J., Zou Y., Ouyang H., Zhao C.C., Zhao L.M. (2018). Fully Bioabsorbable Natural-Materials-Based Triboelectric Nanogenerators. Adv. Mater..

[170] Morel A., Brenes A., Gibus D., Lefeuvre E., Gasnier P., Pillonnet G., Badel A. (2022). A comparative study of electrical interfaces for tunable piezoelectric vibration energy harvesting. Smart Mater. Struct..

[171] Rincón-Mora G.A., Yang S. (2016). Tiny piezoelectric harvesters: Principles, constraints, and power conversion. IEEE Trans. Circuits Syst. I Regul. Pap..

[172] Ghaffarinejad A., Hasani J.Y., Hinchet R., Lu Y., Zhang H., Karami A., Galayko D., Kim S.-W., Basset P. (2018). Aconditioning circuit with exponential enhancement of output energy for triboelectric nanogenerator. Nano Energy.

[173] Coustans M., Krummenacher F., Kayal M. (2019). A Fully Integrated 60 mV cold-start circuit for single coil DC–DC boost converter for thermoelectric energy harvesting. IEEE Trans. Circuits Syst. II Express Briefs.

[174] Long Z., Li P., Chen J., Chung H.S.-H., Yang Z. (2022). Self-Powered Single-Inductor Rectifier-Less SSHI Array Interface with the MPPT Technique for Piezoelectric Energy Harvesting. IEEE Trans. Ind. Electron..

[175] Wang J., Chen Z., Li Z., Jiang J., Liang J., Zeng X. (2022). Piezoelectric energy harvesters: An overview on design strategies and topologies. IEEE Trans. Circuits Syst. II Express Briefs.

[176] Chandrarathna S.C., Graham S.A., Ali M., Yu J.S., Lee J.-W. (2022). An efficient power management system using dynamically configured multiple triboelectric nanogenerators and dual-parameter maximum power point tracking. Adv. Energy Mater..

[177] Wang X., Xia Y., Shi G., Xia H., Chen M., Chen Z., Ye Y., Qian L. (2021). A novel MPPT technique based on the envelope extraction implemented with passive components for piezoelectric energy harvesting. IEEE Trans. Power Electron..

